# A Model for Phylogenetic Chemosystematics: Evolutionary History of Quinones in the Scent Gland Secretions of Harvestmen

**DOI:** 10.3389/fevo.2017.00139

**Published:** 2017-11-17

**Authors:** Günther Raspotnig, Miriam Schaider, Petra Föttinger, Axel Schönhofer

**Affiliations:** 1 Institute of Zoology, University of Graz, Graz, Austria; 2 Research Unit of Osteology and Analytical Mass Spectrometry, University Children’s Hospital, Medical University Graz, Graz, Austria; 3 Institute of Zoology, Johannes Gutenberg University, Mainz, Germany

**Keywords:** opiliones, benzoquinones, naphthoquinones, chemical defense, exocrine secretion

## Abstract

By the possession of unique exocrine scent glands, Opiliones (harvestmen) arise as a perfect model for studies on the evolutionary history of secretion chemistry. Among gland compounds of harvestmen, it is the quinones that represent recurring elements across the secretions of all suborders. Reliable data on quinone-distribution, however, is only known for Laniatores (benzoquinones) and Cyphophthalmi (naphthoquinones). We here unraveled the quinone-distribution across scent gland secretions of the third large harvestman suborder, the Palpatores (= Eu- and Dyspnoi): Naphthoquinones were found in phalangiid Eupnoi across all subfamilies as well as in nemastomatid (and at least one ischyropsalid) Dyspnoi. Benzoquinones (1,4-benzoquinone) were restricted to a small entity within Eupnoi, namely platybunine Phalangiidae, probably misplaced Gyantinae (currently Sclerosomatidae) and *Amilenus* (incertae sedis). Our findings, combined with data from Laniatores and Cyphophthalmi, allow evaluation of a comprehensive chemosystematic model for Opiliones for the first time. Evolutionary scenarios imply naphthoquinones as scent gland compounds of common ancestry, having evolved in an early harvestman ancestor and present in cyphophthalmids and palpatoreans, but lost in laniatoreans. Benzoquinones evolved later and independently at least twice: once in the secretions of gonyleptoid Laniatores (alkylated benzoquinones), and a second time in a lineage of phalangiid Eupnoi (1,4-benzoquinone).

## Introduction

While chemosystematics is a widely accepted discipline in plant systematics, a comparatively low number of chemosystematic studies has been conducted for animals. Moreover, most of these studies have a “chemotaxonomic” focus, i.e., they use specific body surface-derived chemical characters to discriminate between similar or cryptic species. In arthropods, such characters typically comprise cuticular hydrocarbons that represent mixtures of products from various small glands of the epidermis, and contribute in building-up body surface lipid layers. By contrast, chemosystematics—or better “phylogenetic chemosystematics”—is different: it aims to trace the evolutionary history of compounds or compound classes in the secretions of homologous glands, thus trying to explain diversification events in secretion chemistry as well as the extant chemical diversity of glandular secretions in the taxon under consideration. Phylogenetic chemosystematics is clearly limited to taxa possessing homologous gland systems. Such taxa are rare, and in most cases, these represent small sub-entities of a larger taxonomic group only. For instance, when referring to the exocrine glands of beetles—the largest insect order—many different, non-homologous glands can be distinguished. The abdominal glands of the 40,000 species-comprising beetle family Staphylinidae, for instance, are considered to have evolved many times independently (Araujo, 1978; Dettner, 1987), and in strict sense, chemosystematic studies can only be conducted within each of these types, whereas the prerequisites for chemosystematics across all staphylinids is *de facto* not given.

However, a few homologous glandular systems are present across larger taxa: one example is the repugnatorial glands of Diplopoda (e.g., Shear, 2015). Well-known examples for insects include the frontal glands of Isoptera (Prestwich, 1983; Šobotnik et al., 2010) and the pygidial glands of adephagan beetles (Dettner, 1987); examples for arachnids are the oil glands of sarcoptiform mites (e.g., Raspotnig, 2010; Raspotnig et al., 2011). One particularly interesting homologous gland system in arachnids is the scent glands of harvestmen. These constitute paired prosomal glands representing an important synapomorphy of the order Opiliones (e.g., Martens, 1978; Raspotnig, 2012; Raspotnig et al., 2015a). Scent glands are consistently present across all four harvestman suborders (Cyphophthalmi, Eupnoi, Dyspnoi, and Laniatores), and indeed in each of the 6,500 species hitherto described. Scent glands are mainly known for the production of noxious, often distinctly scented secretions, being repellent against predators, even though additional functions, such as antimicrobial and communicative roles, have been discussed (Machado et al., 2002; Schaider and Raspotnig, 2009).

With respect to the highly-diversified and clearly taxon-specific composition of secretions, scent glands represent an important model for understanding and reconstructing chemical traits across an ancient and homologous exocrine system (Hara et al., 2005; Raspotnig, 2012): Considering their ubiquity in harvestmen, it is likely that scent glands already existed before the cladogenesis of harvestmen into the currently recognized suborders. Thus, according to time estimations of opilionid phylogeny, these glands and their secretions may look back to an evolutionary history of more than 350 mya (e.g., Dunlop et al., 2003; Dunlop, 2007; Garwood et al., 2011, 2014). The acquisition, possession, and maintenance of scent glands over such a long period probably played a crucial role in the evolutionary success of harvestmen. From their initial acquisition in a hypothetical harvestman ancestor until today, the chemistry of scent gland secretions has enormously diversified, reflecting the sum of a myriad of specific adaptations over time to particular environmental conditions. From previous studies we know that the composition of secretions clearly characterizes different opilionid lineages. For instance, and very roughly, alkaloids and terpenes appear characteristic for travunioid Insidiatores, phenolics for lower Grassatores, and a number of acyclic compounds (mainly ketones) as well as quinones have been reported from Cyphophthalmi, Eupnoi, Dyspnoi, and Laniatores (e.g., Eisner et al., 1977; Roach et al., 1980; Duffield et al., 1981; Ekpa et al., 1984; Raspotnig et al., 2011, 2014, 2015a,b; Raspotnig, 2012; Wouters et al., 2013).

However, it is the quinones and their derivatives that constitute a most conspicuous class of scent gland components. In general, quinones belong to effective and widely used defensive compounds across Arthropoda (e.g., Blum, 1981), best known from the repulsive spray of bombardier beetles (e.g., Eisner et al., 2006) and the quinonic defense of millipedes (e.g., Eisner et al., 1978). In Opiliones, at least two major taxonomic groups have already been recognized as quinone-producers: Cyphophthalmids discharge naphthoquinones as major secretion components whereas alkylated benzoquinones are considered characteristic for higher Laniatores (Gonyleptoidea). While the distribution of quinones in these two groups is fairly well documented and conclusive (Raspotnig, 2012; Raspotnig et al., 2015a), quinone-production and distribution is almost unknown for suborders Eupnoi and Dyspnoi. Generally, chemical data for the secretions of only a handful of eupnoan and dyspnoan species is available (Wiemer et al., 1978; Ekpa et al., 1985; Raspotnig et al., 2014, 2015b), and these do not indicate ubiquitous, but sporadic quinone-occurrence in a distinct taxonomic pattern. However, this pattern in Eu- and Dyspnoi appears to be highly convoluted with quinone-occurrence being restricted to particular subgroups. Such a taxonomic pattern has possibly arisen from multiple events of quinone regression and re-acquisition. In these terms, quinones certainly constitute an important part of the scent gland-inventory in eu- and dyspnoans as well, and thus, quinones emerge as a recurrent, characteristic element of the scent gland secretions of Opiliones-a kind of chemical “guiding line” in opilionid chemosystematics.

Just recently, a pioneer chemosystematic approach, aiming to elucidate the evolutionary history of benzoquinones in the Laniatores was published, demonstrating that alkylated benzoquinones are a synapomorphic character of higher Laniatores, derived from the ancestral condition of phenolic compounds of lower Laniatores (Caetano and Machado, 2013; Raspotnig et al., 2015a).

We here present a more comprehensive approach providing evidence that quinones are a heterogenous class of scent gland compounds. We show that quinones represent different evolutionary traits that have been invented, maintained, modified or reduced in the course of the evolution of secretions in Opiliones. In detail, our approach was 2 fold: (1) on the one hand, we performed a chemical screening for quinones across the secretions of eupnoan and dyspnoan harvestmen, focusing on European as well as North American taxa, to provide a representative data set on quinone-distribution in chemically understudied Palpatores; (2) on the other hand, we aimed to propose a comprehensive model for quinone-evolution in scent gland secretions that logically explains quinone-distribution across extant Opiliones.

## Materials and Methods

### Species Collection

A total of 2,830 adult individuals, representing 72 species from 31 genera to 8 families and covering the majority of Central European and some North American genera of Palpatores were collected (details in Supplementary Tables 1, 2). Scent gland secretions were extracted and screened for the presence of benzo-, naphtho-, and anthraquinones. For most species, individuals of several populations were investigated. All individuals are currently stored in the collection of the Institute of Zoology in Graz, Austria.

### Extraction of Secretions

Generally, secretion extracts of single individuals were prepared. Scent gland secretions were obtained by the following methods. (i) Whole body extraction: By this method, secretion was directly discharged into the solvent. Whole body extraction was done in 25–250 µl hexane (depending on size of the specimen) and lasted until secretions were exhaustively extracted from scent gland reservoirs. For Eupnoi, an exhaustive extraction of scent gland secretion was already achieved after 30 min, and prolonged extraction times did not increase amounts of extracted secretion any more. For the particular situation in Dyspnoi (glands hidden and covered by an atrium: see Schaider and Raspotnig, 2009), exhaustive extraction of secretion needed 4 h. After 4 h no further increase of amounts of secretion in extracts could be noticed. Whole body extraction was the simple standard extraction method for all species, but was particularly suited for species that quickly released secretions or that released secretions in form of sprays (such as some phalangiids and many sclerosomatids), as well as for small-sized species (e.g., most Nemastomatinae). (ii) As an additional method, discharged liquid secretion from single individuals was adsorbed on filter paper pieces (2 × 2 mm), directly after induction of discharge by mechanical disturbance. Secretion-loaded filter paper pieces were transferred into 50 µl of hexane and extracted for 30 min. This method of direct sampling of secretion was suitable for many Eupnoi, was applied to confirm the scent gland origin of certain compounds and has already been described in a previous paper in detail (Raspotnig et al., 2015b). The method could not be applied for Dyspnoi (that are reluctant to emit secretion). (iii) A further additional method was applied in case of gaseous emission (e.g., for Gyantinae): scenting individuals were placed in pre-cleaned glass vials, and a SPME-fiber (CAR/PDMS, Supelco, Austria) was inserted through a silicon/PTFE-septum (adsorption time was 15 min). (iv) Particularly for species where additional methods (ii and iii) for direct sampling did not work, live specimens were frozen at −20°C, and glands were dissected and subsequently extracted. This was necessary for some Dyspnoi, for *Amilenus*, and a few phalangiids (e.g., *Megabunus* spp.). Methods (ii–iv) for direct secretion sampling were mainly applied to confirm the scent gland origin of compounds detected by whole body extraction, and more than one method was applied for most species.

### Chemical Analysis

Crude extracts were analyzed by gas chromatography—mass spectrometry, using a Trace GC2000 (Thermo, Austria), equipped with a ZB-5 capillary column (30 m × 0.25 mm i.d., × 0.25 μm film thickness (Phenomenex, Germany) at a helium flow of 1.2 ml min^−1^. The temperature of the GC oven was raised from 50°C (1 min) to 300°C at 10°C min^−1^, then held for 5 min at 300°C. The GC was coupled to a DSQ I MS (ion source at 200°C; transfer line at 310°C). All mass spectra were recorded in EI mode. Components adsorbed on SPME-fibers were thermally desorbed in the hot GC injector at 240°C, and analyzed as described above. Retention indices (RI) were calculated using an alkane standard mix according to the formula RI_x_ = 100n_0_ + (100t_x_−100tn_0_)/(tn_1_−tn_0_), with x: target compound; t_x_: retention time of target compound; n_0_: number of carbon atoms in the alkane directly eluting before x; tn_0_: retention time of alkane directly eluting before x; tn_1_: retention time of alkane directly eluting after × (Van den Dool and Kratz, 1963).

### Reference Compounds

6-Methyl-1,4-naphthoquinone was prepared based on a procedure described by Bruce and Thomson (1952). As oxidizing reagent we used CAN (cerium IV ammonium nitrate). 4-Chloro-1,2-naphthoquinone was synthesized from 2,4-dichloro-1-naphthol in benzene using lead(IV)acetate as oxidizing reagent as recently described (Raspotnig et al., 2005). An alkane standard (a mix of C9-C36 n-alkanes), all reagents as well as authentic references for 1,4-benzoquinone, 1,4-naphthoquinone, 1,4-naphthalenediol, 2-methoxy-1,4-naphthoquinone, 2-methyl-9,10-anthraquinone, 1,2-dimethyl-9,10-anthraquinone, 1,4-dimethyl-9,10-anthraquinone, and 2,3-dimethyl-9,10-anthraquinone were purchased from Sigma-Aldrich (Austria).

### Ancestral Character State Reconstruction (ASR)

The phylogenetic tree herein shown is a combination of published trees for particular groups and reflects the current state of harvestman systematics. Trees for particular groups were recently published by Giribet et al. (2010), Hedin et al. (2012a), Schönhofer (2013), and Groh and Giribet (2015). Ancestral character state reconstruction was conducted in Mesquite Version 3.04 (Maddison and Maddison, 2015), mapping distinct characters (naphthoquinones, benzoquinones) under an unordered maximum parsimony regime using a stepmatrix, making gains twice as difficult as losses. This particular method has recently been described and optimized for chemosystematic purposes (Raspotnig et al., 2015a): the method takes into account that newly acquired compounds required a hierarchical multistep machinery to their synthesis (=difficult), whereas the loss of a compound may happen in one step by the inactivation of a single enzyme (=easy).

## Results

### Identification of Quinones

**Figure 1:**
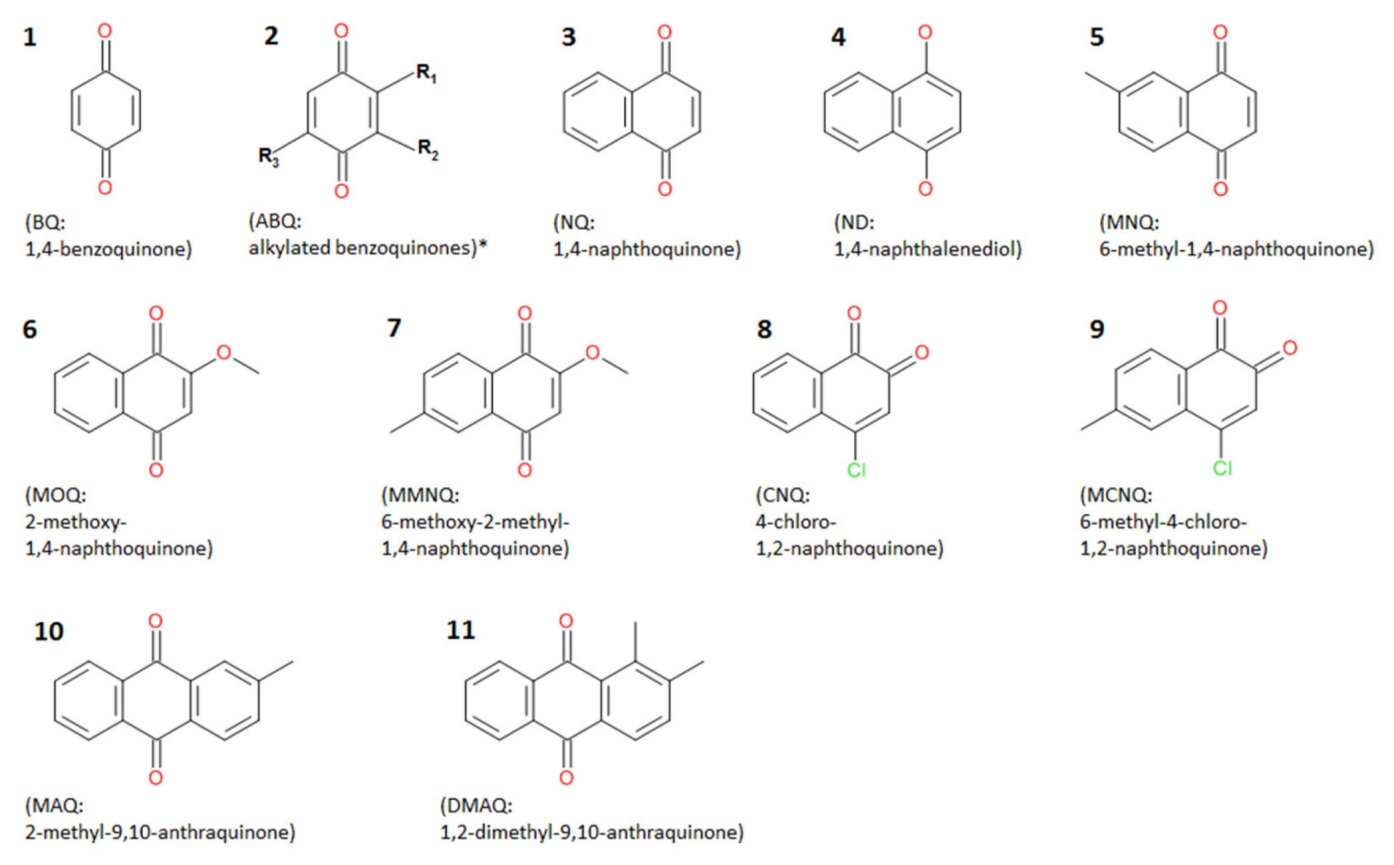
Chemical formulas of quinonic compounds found in the secretions of palpatorean harvestmen. Numbers refer to chemical characters in Figure 3. *Alkylated benzoquinones may contain different substituents: R_1_ = H or CH_3_ or C_2_H_5_ or C_3_H_7_; R_2_ = H or CH_3_; R_3_ = H or CH_3_ or C_2_H_5_ (according to Hara et al., 2005 and Gnaspini and Hara, 2007).

Among a multitude of components found in the extracts of eupnoans and dyspnoans herein analyzed (will be published elsewhere), nine quinonic compounds were detected, namely 1,4-benzoquinone (BQ), 1,4-naphthoquinone (NQ), 1,4-naphthalendiol (ND), 6-methyl-1,4-naphthoquinone (MNQ), 2-methoxy-1,4-naphthoquinone (MOQ), 2-methoxy-6-methyl-1,4-naphthoquinone (MMOQ), 4-chloro-1,2-naphthoquinone (CNQ), 2-methyl-9,10-anthraquinone (MAQ), and 2,3-dimethyl-9,10-anthraquinone (DMAQ) (Figure 1). All compounds were already known to us from previous studies (e.g., Raspotnig et al., 2005, 2010, 2015b) and were readily identified by comparison of GC-MS data to authentic reference compounds (analytical data in Table 1). For MMOQ, no authentic reference was available and the position of substituents (methoxy-group in position 2; methyl-group in position 6) is tentatively proposed in analogy to the substitution pattern of the remaining naphthoquinones (e.g., MNQ, MOQ). DMAQ—previously proposed to be a 2,3-dimethyl-substituted anthraquinone (Raspotnig et al., 2010)—revealed a retention time different from authentic 2,3-DMAQ (measured RI of 2,3-DMAQ: 2318). A comparison of DMAQ with two further isomers (1,4-DMAQ and 1,2-DMAQ) showed full correspondence to authentic 1,2-DMAQ. Co-injection of an harvestman-extract containing DMAQ and authentic 1,2-DMAQ resulted in a single sharp peak at *RI* = 2,277 without shoulders.

### Source of Quinones

When mechanically disturbed (e.g., grasped at a leg or squeezed moderately), all eupnoan naphthoquinone-producers (Table 2) visibly emitted their secretions as liquids directly oozing out from ozopores (e.g., *Phalangium*) or as directed forceful jets (e.g., *Rilaena*). In these cases, adsorption of discharged liquids on filter papers was possible, and extracts of secretion-loaded filter papers showed naphthoquinone-patterns indistinguishable from whole body extracts of conspecific individuals. Filter papers loaded with jets from the ozopores of *Rilaena triangularis* contained both 1,4-naphthoquinone and 1,4-benzoquinone, just as found in extracts of whole bodies. For species of the genus *Opilio* (Opilioninae), no conclusive data on scent gland-associated naphthoquinone-production could be obtained. However, the discharge of droplets or at least liquids from ozopores was observed for all included species except for *O. dinaricus*.

By contrast, none of the dyspnoan naphthoquinone-producers (*Nemastoma*, *Carinostoma*, *Paranemastoma*, *Histricostoma, Hesperonemastoma*) emitted any visible liquid from their ozopores nor could any smell be recognized. Nevertheless, whole body extracts of all these species contained naphthoquinones (Table 3). The amounts of naphthoquinones in such extracts were generally low (traces only) but increased by the time of extraction, reaching a maximum after about 4 h in heated (50°C) solvent. Glandular sacs excised from frozen individuals in all the nemastomatines mentioned above appeared to be slightly reddish, and extracts of excised glands exhibited large amounts of naphthoquinones.

Upon mechanical disturbance, all benzoquinone-producers (*Megabunus, Platybunus, Amilenus, Gyas*) with the exception of *Rilaena*, released a characteristic, unpleasant odor reminiscent of 1,4-benzoquinone, but no liquids. Mechanical triggers for scent emission were grasping the legs or gently squeezing the body. The secretion was emitted as a gaseous puff that faded out within a few seconds. For some species, such as *M. armatus*, only a faint odor was noticed at best, corresponding to low amounts of 1,4-benzoquinone as recorded from whole body extracts of this species. Other species, such as *Gyas* spp., released clearly noticeable quinonic scents. In order to prove the scent gland origin of gaseous 1,4-benzoquinone emission, we placed small filter papers above the ozopores of scenting individuals, thus exposing these filter papers directly to the emitted scent. Extracts of such filter papers contained small amounts of 1,4-benzoquinone. Moreover, 1,4-benzoquinone from scenting individuals placed in a closed jar could be absorbed on SPME-fibers, as most easily demonstrated for species of *Gyas*. Dissection of scent glands in all these benzoquinone-producing species revealed glands filled with a brownish, viscous paste. Finally, subsequent extraction of excised glands in dichloromethane confirmed the presence of 1,4-benzoquinone.

### Taxonomic Distribution of Quinones across the Eupnoi

Quinonic secretions appeared to be restricted to family Phalangiidae in representatives of which either naphthoquinones or benzoquinones were detected (Table 2). Naphthoquinones (NQ + MNQ) occurred in all four phalangiid subfamilies, Phalangiinae, Platybuninae, Opilioninae, and Oligolophinae, showing a distinct taxonomic pattern of distribution. While all phalangiines investigated (*Phalangium, Rilaena*) possessed naphthoquinones, the secretions of only a distinct part of the Platybuninae (only *Lophopilio*, not *Platybunus*, *Megabunus*), Oligolophinae (only *Lacinius ephippiatus, L. horridus, Oligolophus tridens*, not *L. dentiger, Mitopus morio)*, and Opilioninae (only O. *ruzickai,* not other *Opilio* spp. nor *Egaenus)* were naphthoquinonic. Data for *Opilio*, however, needs confirmation (only a few individuals of *Opilio* spp. were available for investigation).

Benzoquinones (BQ) were detected in the secretions of all platybunine Phalangiidae investigated, except for *Lophopilio*. In detail, BQ was present in the secretions of all five Central European species of *Megabunus* (*M. lesserti, M. armatus,* *M. bergomas, M. rhinozeros, M. vignai*) and in *Platybunus* (*P. bucephalus*). The Central European platybunine genus *Lophopilio*, represented by *L. palpinalis*, conspicuously lacked benzoquinone (but showed NQ + MNQ as indicated above). BQ (together with NQ) was found in the scent gland secretion of the phalangiine *Rilaena triangularis*, hence representing the only example investigated to exhibit both benzo- and naphthoquinones.

BQ was also detected in extracts of *Amilenus aurantiacus* (“*Dicranopalpus*-group”). No conclusive chemical data could be gained for *Dicranopalpus gasteinensis*. Moreover, BQ occurred in some taxa currently grouped with the second large eupnoan family, Sclerosomatidae. The compound was present in secretions of both Austrian species of *Gyas* (*G. titanus* and *G. annulatus*) which according to Pinto da Rocha and Giribet (2007) belong to subfamily Gyantinae within Sclerosomatidae but are assigned to Phalangiidae by Hedin et al. (2012b).

All other Sclerosomatidae herein studied (five species of *Leiobunum*, four species of *Astrobunus*, and two species of *Nelima*) exhibited secretions devoid of naphtho- and benzoquinones. Accordingly, quinones were not detected for American Protolophidae, such as *Protolophus niger*, and *P. singularis*.

### Taxonomic Distribution of Quinones across the Dyspnoi

Only naphtho- and anthraquinonic, but not benzoquinonic secretions were found in Dyspnoi. NQs were detected in representatives of superfamily Troguloidea and less often in Ischyropsalidoidea (Table 3). In Troguloidea, NQ and MNQ were particularly characteristic for nemastomatine Nemastomatidae, i.e., for species of genera *Carinostoma, Nemastoma, Histricostoma,* and *Paranemastoma*. Small amounts of NQ and MNQ were also found in extracts of *Centetostoma* and *Mediostoma*, but this data relies on a few individuals only and needs confirmation. In species of *Paranemastoma,* NQ and MNQ were accompanied by small or trace amounts of ND, MOQ, and MMOQ. The latter two compounds were also recorded for certain *Nemastoma (*e.g., *N. triste)* and for *Carinostoma* spp. (Table 3). For the second dyspnoan superfamily, Ischyropsalidoidea, NQ + MNQ were detected in species of *Hesperonemastoma* (assigned to Sabaconidae by (Giribet et al., 2010) or to Ceratolasmatidae by (Shear, 1986), respectively). Unexpectedly, several *Nemastoma*-species (*N. schuelleri*, *N. triste*, and a subspecies of the new and still undescribed *N. bidendatum*-ssp. complex), all species of *Carinostoma* as well as *Hesperonemastoma* (Ischyropsalidoidea) contained small amounts of CNQ as well. Anthraquinones (MAQ, DMAQ) were found characteristic of representatives of *Paranemastoma* with traces of these compounds sporadically found in certain *Nemastoma* as well (Table 3). Regarding the Nemastomatinae, only the secretions of *Mitostoma* were devoid of quinones.

Quinonic secretions were neither found in the Ortholasmatinae (Nemastomatidae: genera *Ortholasma* and *Dendrolasma* analyzed) nor in the troguloid families, Trogulidae (species of *Trogulus* examined) and Dicranolasmatidae (*Dicranolasma scabrum* examined). Quinones were not detected in any ischyropsalidoidid taxon apart from *Hesperonemastoma* (genera *Ischyropsalis* and *Taracus* examined).

### Evolutionary History of Naphtho- and Benzoquinones

**Figure 2:**
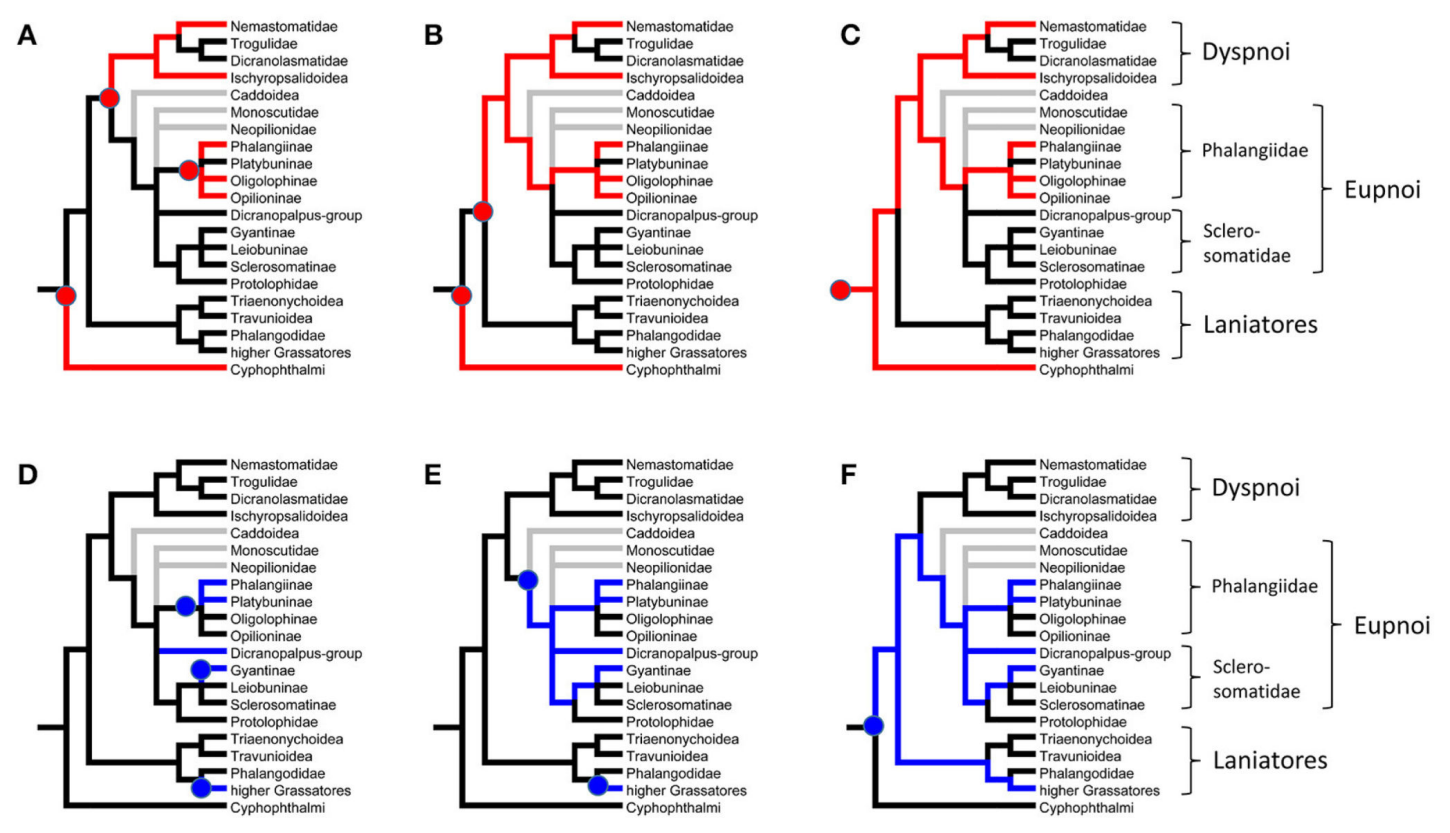
Eligible hypotheses for the evolution of quinones in the scent glands of Opiliones. In Red: Naphthoquinones. **(A)** Multiple independent evolution in Cyphophthalmi, Dyspnoi, and phalangiid Eupnoi. **(B)** Independent evolution in Cyphophthalmi and Palpatores. **(C)** Common ancestry. In Blue: Benzoquinones. **(D)** Multiple independent evolution in Gonyleptoidea (higher Grassatores), sclerosomatid Gyantiinae, *Dicranoplalpus*-group, and Phalangiidae. **(E)** Independent evolution in Palpatores and Gonyleptoidea (higher Grassatores). **(F)** Common ancestry.

**Figure 3:**
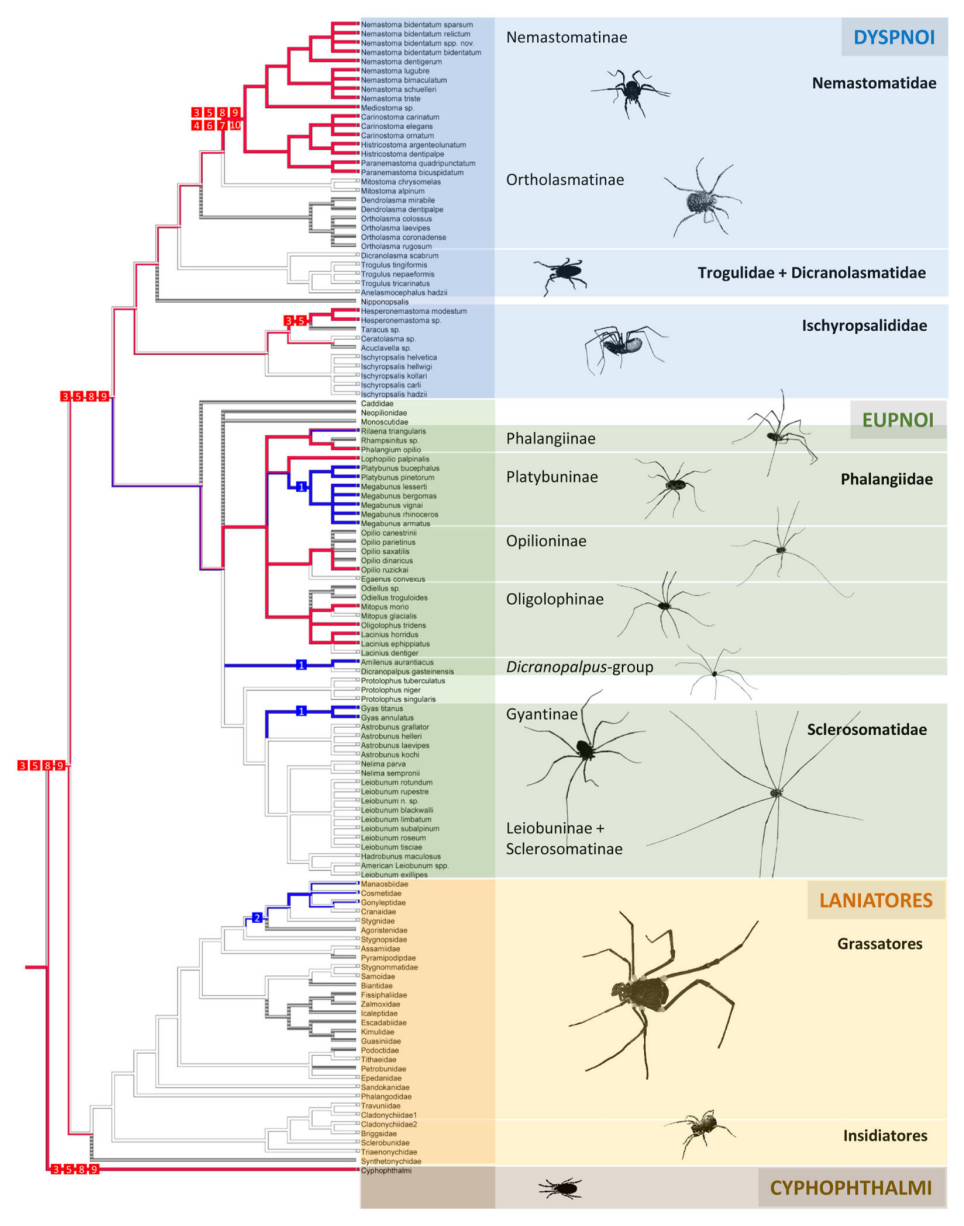
Evolutionary history of naphthoquinones (red) and benzoquinones (blue) in the scent glands of Opiliones as evidenced by ancestral character state reconstruction (ASR). Numbers 1–11 (= chemical characters) refer to compounds from Figure 1. ASR was based on a maximum parsimony reconstruction of discrete characters using a stepmatrix in which gains are twice less probable as losses, as proposed for chemosystematics (Raspotnig et al., 2015a). Harvestman phylogeny has been combined from literature. The overall relationships of suborders as pictured [(Cyphophthalmi) (Laniatores) (Eupnoi + Dyspnoi)] is meanwhile generally accepted (e.g., Hedin et al., 2012a; Fernández et al., 2017). For Eupnoi, for which no representative molecular genetic study exists, the common division into two superfamilies (Caddoidea, Phalangioidea) is retained. Phalangioidea is divided into 5 groups as in Pinto da Rocha and Giribet (2007)—Neopilionidae, Monoscutidae, Phalangiidae, Sclerosomatidae, and the *Dicranopalpus* group—which are shown as a polytomy. Phalangiidae and Sclerosomatidae are divided into subfamilies according to Pinto da Rocha and Giribet (2007); subfamilies are again shown as polytomies. For Dyspnoi, we referred to the phylogeny proposed by Schönhofer (2013), because it includes all classical dyspnoan genera. It differs from dyspnoan phylogeny from Fernández et al. (2017) who include Acropsopilionidae as an early-branching dyspnoan taxon. For the results of our ASR, this makes no difference.

With the already existing data of quinone occurrence in Cyphophthalmi and Laniatores (Table 4), the data on palpatoreans were compiled to a comprehensive data-base on quinone-distribution across the Opiliones. From this basal matrix, six eligible scenarios for naphtho- and benzoquinone evolution in opilionids arise: (i) naphthoquinones might have evolved three times independently, namely once in Cyphophthalmi, a second time in Dyspnoi (nemastomatines + *Hesperonemastoma*), and a third time in a lineage of phalangiine Eupnoi (Figure 2A). (ii) Alternatively, naphthoquinones might have been acquired twice (once in Cyphophthalmi, once in Palpatores) (Figure 2B), or (iii) might share a common ancestry in Opiliones (Figure 2C). Comparably, benzoquinones (iv) might have been acquired independently in platybunine Eupnoi (plus *Amilenus*), in gyantiine sclerosomatids, and in laniatoreans (Figure 2D); (v) might have been acquired independently in palpatoreans and in Laniatores (Figure 2E), or, (vi) might share a single origin in a common ancestor of Palpatores and Laniatores (Figure 2F). To test these hypotheses, the evolutionary history of characters “naphthoquinones” and “benzoquinones” was traced across all Opiliones using a comprehensive, combined phylogeny of harvestmen. Eventually, ASR (ancestral character state reconstructions) of naphthoquinones (shown as red lines in Figure 3) implied a single acquisition of these compounds in a common opilionid ancestor and their multiple convergent regression in various lineages of Eu- and Dyspnoi as well as complete naphthoquinone-reduction in early laniatoreans. By contrast, ASR of benzoquinones indicated that they evolved at least twice: once in a lineage of Phalangiidae (including *Amilenus* and gyantiines), and a second time in gonyleptoid Laniatores (blue lines in Figure 3).

## Discussion

### The Harvestman Quinone Network

It has traditionally been considered that alkylated benzoquinones are a basic feature of gonyleptoid Laniatores (Hara et al., 2005; Gnaspini and Hara, 2007) whereas cyphophthalmid secretions may generally contain naphthoquinones (representatives of 3 out of 6 families analyzed: Raspotnig et al., 2005, 2012; Jones et al., 2009). By contrast, quinones in palpatorean secretions were rather regarded as exceptions and quinone distribution across palpatoreans had even remained completely unclear. From the approximately 1,800 species of Eupnoi and 290 species of Dyspnoi, the scent gland secretions of only 13 eupnoans (11 leiobunine Sclerosomatidae, 2 phalangiine Phalangiidae) and four dyspnoans (four nemastomatine Troguloidea) had been studied, and only a few of these actually produced quinones. In detail, Wiemer et al. (1978) firstly reported that *Phalangium opilio* (Eupnoi, Phalangiidae) discharged naphthoquinones. Raspotnig et al. (2015b) found the confamiliar *Rilaena triangularis* to be both a naphtho- and benzoquinone-producer, and Raspotnig et al. (2010, 2014) reported naphtho- and anthraquinones from nemastomatine Dyspnoi as well.

In order to achieve a near-comprehensive, representative picture of quinone production and emission in the Opiliones, we here evaluated data for most Central European and some North American Palpatores. We though admit that our screening is still far from being complete: with respect to Eupnoi, not a single representative of superfamily Caddoidea has yet been analyzed, and for the Phalangioidea, chemical data on members of the smaller families Monoscutidae and Neopilionidae are missing. Regarding the Dyspnoi, the monogeneric East Asian family Nipponopsalididae could not be covered. The same is true for the Acropsopilionidae, which recently have been transferred from Eupnoi to Dyspnoi (e.g., Groh and Giribet, 2015; Fernández et al., 2017). For all other palpatorean families, at least one representative was included into our study.

In our combined data set (Tables 2, 3), quinones emerge as a major chemical class in palpatorean scent gland secretions, filling a gap in the knowledge of quinone-distribution across the secretions of Opiliones. Thus, two major sub-classes of quinones—naphtho- and benzoquinones— characterize harvestmen scent gland secretions, and based on our ASRs, quinones most likely have evolved three times independently. Naphthoquinones share a common ancestry and possibly already evolved in a hypothetical common harvestman ancestor. By contrast, benzoquinones evolved later and at least twice, namely 1,4-benzoquinone in platybunine Eupnoi, and alkylated benzoquinones in higher Laniatores. Such a situation is very unusual, if not unique among arthropods. For the “quinone millipedes,” the prime example of arthropod quinone producers, a single origin of quinones (i.e., benzoquinones) is likely (Bodner and Raspotnig, 2012; Shear, 2015; Bodner et al., 2016). In many other arthropods, the occurrence of quinonic secretions is restricted to small, compact taxonomic entities, indicating a monophyletic origin as well (e.g., in Blattaria, Dermaptera: see Blum, 1981). For Coleoptera, quinones have arisen independently in the secretions of several beetle taxa, such as in Carabidae and Tenebrionidae (Tschinkel, 1972; Francke and Dettner, 2004), but do not characterize the whole order. We hypothesize that the events of quinone evolution as well as their multiple regression in various groups of harvestmen reflect the many instances of adaptation of harvestmen to particular environmental conditions in the course of their evolution. Possibly, in non-quinone producing taxa, such as Sclerosomatidae, an adaptation to a mainly hypergeic life-style promoted quinone-regression in favor of other, more volatile components. In hypergeic Phalangiidae, however, quinones may show a relict pattern, but do not occur as regularly as in edaphic/epigeic cyphophthalmids and higher laniatoreans.

### A Model for Phylogenetic Chemosystematics

We here provide a first comprehensive hypothesis on quinone evolution in the secretions of Opiliones, proposing a coherent phylogenetic chemosystematic model all across a homologous gland system. Our model is based on results from ASR (ancestral character state reconstruction) and relies on mapping the character “quinones” onto a synthetic - and partly simplified - phylogeny of Opiliones. As an overall-schedule for a phylogenetic arrangement of suborders/superfamilies we referred to the “classical opilionid system” (sensu Giribet and Kury, 2007) that pictures basal Cyphophthalmi and monophyletic Palpatores [= Cyphophthalmi (Palpatores + Laniatores)] as most recently re-confirmed by next-generation transcriptome data analysis (Hedin et al., 2012a; Fernández et al., 2017). For detailed phylogenies of distinct groups, we implemented currently accepted phylogenies for Dyspnoi (Schönhofer, 2013), Laniatores (Sharma and Giribet, 2011), and Eupnoi (Pinto da Rocha and Giribet, 2007) into the overall system (for details, see legend of Figure 3).

Interestingly, we discovered chemically-characterized lineages/clades that do not correspond to traditional classifications. For instance, 1,4-benzoquinone most likely characterizes a distinct clade in Phalangiidae that is currently not recognized. We consider 1,4-benzoquinone to have arisen anew in ancestors of platybunine phalangiids. The compound is present in the Central European lineage of platybunines (Starega, 1976) with the exception of *Lophopilio palpinalis*. The conspicuous lack of 1,4-benzoquinone in *Lophopilio* is in agreement with its phalangiine-like scent gland secretion profile (=containing naphthoquinones) and supports the idea of misplacement of *L. palpinalis* in the Platybuninae. The co-occurrence of 1,4-benzoquinone and 1,4-naphthoquinone in the scent gland secretion of *Rilaena triangularis* deserves some attention: though currently assigned to (naphthoquinone-producing) Phalangiinae (e.g., Martens, 1978), *Rilaena* is often listed as a platybunine in literature (e.g., Starega, 1976), and its assignment to either of these two subfamilies is still controversial. Our data provides evidence that *Rilaena* also chemically holds an intermediate position between phalangiines and platybunines, indicating that the phalangiid benzoquinone-lineage may be closely related to naphthoquinone-producing phalangiines. Moreover, *Gyas* and *Amilenus* show 1,4-benzoquinone-rich secretions as well, and we do not believe that this is a result of parallelism. More likely than an independent evolution of benzoquinones in rather closely related genera, the current taxonomic placement of both genera is in need of reconsideration, and *Gyas* and *Amilenus* possibly have to be re-grouped with the benzoquinone-lineage of Phalangiidae. At least for *Gyas*, a recent molecular study already supports this idea, indicating the misplacement of *Gyas* in Sclerosomatidae and proposing its inclusion into Phalangiidae (Hedin et al., 2012b). In a very recent paper, Fernández et al. (2017) also show *Gyas* with Phalangiidae, as sister taxon of *Odiellus*.

We used here a very robust ASR-technique, adjusted to the particular situation in chemosystematics: since compounds are derived from hierarchically-arranged biosynthetic multi-step pathways, the acquisition of a compound needs several steps. Thus, the convergent development of a compound becomes much less likely than its loss (which may be achieved in one step by the inactivation of a single enzyme). To take this particular situation into account, we relied on stepmatrices, making gains more difficult as losses. We just used a “stepmatrix 2” (a gain is twice as difficult as a loss) when reconstructing the characters “NQs” and BQs,” even though certainly more than two biosynthetic steps are involved in NQ- and BQ-synthesis. A similar ASR-technique has recently been applied to reconstruct the evolutionary history of phenols and benzoquinones in the secretions of Laniatores (Raspotnig et al., 2015a).

### The Evolutionary History of Naphthoquinones in Harvestman Secretions

Naphthoquinones have already been shown to prevail the secretions of cyphophthalmids (Table 4). We here prove that NQs characterize a distinct part of the Palpatores as well. In detail, naphthoquinones were detected in the secretions of all four subfamilies of phalangiid Eupnoi (Phalangiinae, Platybuninae, Oligolophinae, and Opilioninae), in most nemastomatine Dsypnoi and in at least one representative of Ischyropsalidoidea (*Hesperonemastoma*). Contrasting the situation in harvestmen, naphthoquinone-rich secretions must be considered rather rare in arthropods. In insects, these compounds are known from some tenebrionid beetles (Tschinkel, 1972) as well as from Thysanoptera (Suzuki et al., 1995, 2004). In the “quinone millipedes” (sensu Eisner et al., 1978), naphthoquinones are usually absent from secretions but were exceptionally reported as trace-components from the exudates of a single spirostreptid diplopod species (Deml and Huth, 2000). For arachnids other than Opiliones, only one report on naphthoquinone-emission in a mite species has so far been published (Sakata et al., 1997). It indeed appears to be exclusive to harvestmen that naphthoquinones are such widely used as exocrine compounds.

There is evidence that NQs represent phylogenetically old compounds in the Opiliones: NQs may characterize Cyphophthalmi as a whole, but are considered subjected to several events of regression in Eupnoi and Dyspnoi. Corroborating a common ancestry, the major compounds involved are in all Opiliones the same, namely 1,4-NQ and 6-MNQ. In addition, some very unusual naphthoquinones, such as chloro-naphthoquinones (CNQs), are shared by cyphophthalmids and a few nemastomatine Dyspnoi. These CNQs have not been found yet in any other arthropod taxon, thus making their parallel evolution in two closely related groups even more unlikely. By contrast, the Laniatores lack NQs completely, which may be explained by an early, radical reduction event in laniatorean ancestors. Such a scenario is illustrated in Figure 2C. Alternative scenarios would involve the much less parsimonious multiple independent evolution of naphthoquinones in the secretions of opilionids (Figures 2A,B) and Palpatores in particular.

### The Evolutionary History of Benzoquinones in Harvestman Secretions

In contrast to naphthoquinones, benzoquinone-distribution across harvestman secretions is more convoluted. Generally, the detection of 1,4-BQ in the secretions of a considerable group of Eupnoi was more than unexpected. So far, data on harvestman secretions mainly supported the paradigm of a deep chemical divergence between Cyphophthalmi/Palpatores (as non-benzoquinone-producers) on the one hand, and the generally benzoquinone-producing higher Laniatores on the other hand (Raspotnig, 2012). First evidence for a possibly much more complex situation in the higher Laniatores was given by Rocha et al. (2011) and Wouters et al. (2013) who found a non-benzoquinone-producing clade within Gonyleptoidea (“clade K92,” as introduced by Caetano and Machado, 2013). We here provide data that proves benzoquinones as a third chemical class in the secretions of eupnoan Palpatores besides already known acycles (e.g., Ekpa et al., 1985; “sclerosomatid compounds” sensu Raspotnig 2012) and (herein specified) naphthoquinones. In detail, scent gland-derived benzoquinones have always been regarded as a synapomorphy of more-derivative grassatorean Laniatores (e.g., Hara et al., 2005; Föttinger et al., 2010), and we indeed believe that the occurrence of benzoquinones in palpatorean and laniatorean secretions is the result of independent evolution. Arguments supporting our view stem from (i) the completely different systematic positions of Eupnoi and Grassatores in all currently recognized phylogenies (e.g., Giribet et al., 1999; Shultz and Regier, 2001), and (ii) the specific differences in the chemistry of palpatorean and laniatorean benzoquinones. While all laniatorean benzoquinones show alkyl-substitution (preferably in 2, 3, and 5 position), exclusively unsubstituted 1,4-benzoquinone was found in the eupnoans studied herein.

These chemical differences may be due to different biosynthetic pathways of benzoquinone production. A number of biosynthetic routes to quinones has been reported from plants, fungi, and diverse microorganisms, whereas data concerning arthropods, particularly regarding the polyketid pathway, are scarce or even missing (Blum, 1987; Morgan, 2004; Pankewitz and Hilker, 2008). According to Meinwald et al. (1966) substituted benzoquinones and unsubstituted 1,4-benzoquinone may be derived from different biosynthetic pathways even in one and the same species. With respect to laniatoreans, alkylated benzoquinones are considered to be synthesized *de novo* from acetate/propionate units (Rocha et al., 2013), leading to a polyketide chain followed by cyclisation, enolyzation, and decarboxylation resulting in a substituted phenol. Phenols are still present in many lower grassatoreans (Föttinger et al., 2010; Shear et al., 2010; Raspotnig et al., 2015a). Eventually, alkylated benzoquinones of higher laniatoreans may be produced by an extension of the pathway to phenols via para-oxidation (Rocha et al., 2013). In our study, however, no phenolic precursors were found, and the biosynthetic route to unsubstituted 1,4-benzoquinone in eupnoans may be different and remains to be investigated.

## Supplementary Material

The Supplementary Material for this article can be found online at: https://www.frontiersin.org/articles/10.3389/fevo.2017.00139/full#supplementary-material

Supplementary Table 1

Supplementary Table 2

## Figures and Tables

**Table 1:** Gas chromatographic – mass spectrometric data to quinones from Palpatores.

Compound	RI	M+ (m/z)	Diagnostic ions (m/z)	Identified as
BQ	917	108	82, 80, 54	1,4-benzoquinone
NQ	1,422	158	130, 104, 102, 76, 75	1,4-naphthoquinone
ND	1,493	160	132, 131, 105, 104, 77, 76	1,4-naphthalenediol
MNQ	1,547	172	157, 144, 118, 116, 115, 90, 89	6-methyl-1,4-naphthoquinone
CNQ	1,604	194/192*	164, 157, 129, 104, 101, 76, 75, 74	4-chloro-1,2-naphthoquinone
MOQ	1,782	188	173, 160, 159, 158, 131, 130, 104, 102, 101, 89, 76	2-methoxy-1,4-naphthoquinone
MMOQ	1,911	202	187, 174, 173, 172, 146, 145, 144, 131, 118, 116, 115, 103, 89, 77	methoxy-methyl-1,4-naphthoquinone**
MAQ	2,134	222	221, 207, 194, 193, 166, 165, 164, 163, 139	2-methyl-9,10-anthraquinone
DMAQ	2,277	236	235, 221, 208, 207, 193, 179, 178, 165	1,2-dimethyl-9,10-anthraquinone

* Isotopic pattern arising from ^35^Cl/^37^Cl.

** The position of the substituents was not determined (see text). Detailed analytical data to compounds are found in Raspotnig et al. (2005, 2010, 2015b).

**Table 2:** Naphtho- and benzoquinone distribution across the secretions of Eupnoi*.

(Super)family	Subfamily	*Species*	NQ	MNQ	MOQ	MMOQ	ND	CNQ	MAQ	DMAQ	BQ	ABQ
**PHALANGIOIDEA**												
Phalangiidae	Phalangiinae	*Phalangium opilio*	■	■	□	□	□	□	□	□	□	□
		*Rilaena triangularis*	■	□	□	□	□	□	□	□	■	□
	Oligolophinae	*Lacinius dentiger*	□	□	□	□	□	□	□	□	□	□
		*Lacinius ephippiatus*	□	■	□	□	□	□	□	□	□	□
		*Lacinius horridus*	□	■	□	□	□	□	□	□	□	□
		*Mitopus morio*	□	□	□	□	□	□	□	□	□	□
		*Oligolophus tridens*	□	■	□	□	□	□	□	□	□	□
		*Odiellus* sp.	?	?	?	?	?	?	?	?	?	?
	Platybuninae	*Lophopilio palpinalis*	■	■	□	□	□	□	□	□	□	□
		*Megabunus armatus*	□	□	□	□	□	□	□	□	■	□
		*Megabunus bergomas*	□	□	□	□	□	□	□	□	■	□
		*Megabunus lesserti*	□	□	□	□	□	□	□	□	■	□
		*Megabunus rhinoceros*	□	□	□	□	□	□	□	□	■	□
		*Megabunus vignai*	□	□	□	□	□	□	□	□	■	□
		*Platybunus bucephalus*	□	□	□	□	□	□	□	□	■	□
	Opilioninae	*Egaenus convexus*	□	□	□	□	□	□	□	□	□	□
		*Opilio canestrinii*	□	□	□	□	□	□	□	□	□	□
		*Opilio dinaricus*	□	□	□	□	□	□	□	□	□	□
		*Opilio parietinus*	□	□	□	□	□	□	□	□	□	□
		*Opilio ruzickai*	?	?	?	?	?	?	?	?	-	-
		*Opilio saxatilis*	□	□	□	□	□	□	□	□	□	□
Protolophidae		*Protolophus niger*	□	□	□	□	□	□	□	□	□	□
		*Protolophus singularis*	□	□	□	□	□	□	□	□	□	□
Sclerosomatidae	Leiobuninae	*Leiobunum blackwalli*	□	□	□	□	□	□	□	□	□	□
		*Leiobunum limbatum*	□	□	□	□	□	□	□	□	□	□
		*Leiobunum roseum*	□	□	□	□	□	□	□	□	□	□
		*Leiobunum rotundum*	□	□	□	□	□	□	□	□	□	□
		*Leiobunum rupestre*	□	□	□	□	□	□	□	□	□	□
		*Leiobunum subalpinum*	□	□	□	□	□	□	□	□	□	□
		*Leiobunum* sp. nov.	□	□	□	□	□	□	□	□	□	□
		*Nelima sempronii*	□	□	□	□	□	□	□	□	□	□
		*Nelima troglodytes*	□	□	□	□	□	□	□	□	□	□
	Sclerosomatinae	*Astrobunus dinaricus*	□	□	□	□	□	□	□	□	□	□
		*Astrobunus helleri*	□	□	□	□	□	□	□	□	□	□
		*Astrobunus kochi*	□	□	□	□	□	□	□	□	□	□
		*Astrobunus laevipes*	□	□	□	□	□	□	□	□	□	□
	Gyantinae	*Gyas annulatus*	□	□	□	□	□	□	□	□	■	□
		*Gyas titanus*	□	□	□	□	□	□	□	□	■	□
	*Dicranopalpus*-group	*Amilenus aurantiacus*	□	□	□	□	□	□	□	□	■	□
		*Dicranopalpus gasteinensis*	□	□	□	□	□	□	□	□	?	□

* Only representatives of Phalangioidea considered. Representatives of the second superfamily Caddoidea (exclusively comprising non-European species) were not available for this study.

NQ, (1,4-naphthoquinone); MNQ, (6-methyl-1,4-naphthoquinone); MOQ, (2-methoxy-1,4-naphthoquinone); MMOQ, (methoxy-methyl-1,4-naphthoquinone); ND, (1,4-naphththalendione); CNQ, (chloro-naphthoquinones); MAQ, (2-methyl-9,10-anthraquinone); DMAQ, (1,2-dimethyl-9,10-anthraquinone); BQ, (1,4-benzoquinone); ABQ, (alkyl-substituted benzoquinones). ■= detected, □= not detected, ? = no conclusive data, - = data missing.

**Table 3:** Naphtho- and benzoquinone distribution across the secretions of Dyspnoi.

(Super)family	Subfamily	*Species*	NQ	MNQ	MOQ	MMOQ	ND	CNQ	MAQ	DMAQ	BQ	ABQ
**TROGULOIDEA**												

Dicranolasmatidae		*Dicranolasma scabrum*	□	□	□	□	□	□	□	□	□	□
		*Dicranolasma soerensii*	□	□	□	□	□	□	□	□	□	□
Nemastomatidae	Nemastomatinae	*Carinostoma carinatum*	■	■	□	□	□	□	□	□	□	□
		*Carinostoma elegans*	■	■	□	□	□	□	□	□	□	□
		*Carinostoma ornatum*	■	■	□	□	□	□	□	□	□	□
		*Centetostoma* sp.	■	■	□	□	□	□	□	□	□	□
		*Histricostoma argenteolunatum*	■	■	□	□	□	□	□	□	□	□
		*Histricostoma dentipalpe*	■	■	□	□	□	□	□	□	□	□
		*Mediostoma humerale*	■	■	□	□	□	□	□	□	□	□
		*Mitostoma chrysomelas*	□	□	□	□	□	□	□	□	□	□
		*Nemastoma b. bidentatum*	■	■	□	□	□	□	□	□	□	□
		*Nemastoma b. relictum*	■	■	□	□	□	□	□	□	□	□
		*Nemastoma b. sparsum*	■	■	□	□	□	□	□	□	□	□
		*Nemastoma bidentatum* ssp. nov.*	■	■	◩	□	□	◩	◩	□	□	□
		*Nematoma bimaculatum*	?	?	?	?	?	?	?	?	?	?
		*Nemastoma dentigerum*	■	■	□	□	□	◩	□	□	□	□
		*Nemastoma lugubre*	■	■	□	□	□	◩	□	□	□	□
		*Nemastoma schuelleri*	■	■	□	□	□	◩	□	□	□	□
		*Nemastoma triste*	■	■	□	◩	□	◩	◩	□	□	□
		*Paranemastoma bicuspidatum*	■	■	■	■	■	□	■	■	□	□
		*Paranemastoma quadripunctatum*	■	■	■	■	■	□	■	■	□	□
	Ortholasmatinae	*Dendrolasma dentipalpe*	□	□	□	□	□	□	□	□	□	□
		*Dendrolasma mirabile*	□	□	□	□	□	□	□	□	□	□
		*Ortholasma colossus*	□	□	□	□	□	□	□	□	□	□
		*Ortholasma coronadense*	□	□	□	□	□	□	□	□	□	□
		*Ortholasma levipes*	□	□	□	□	□	□	□	□	□	□
		*Ortholasma rugosum*	□	□	□	□	□	□	□	□	□	□
Trogulidae		*Trogulus tingiformis*	□	□	□	□	□	□	□	□	□	□
		*Trogulus* sp.	□	□	□	□	□	□	□	□	□	□

**ISCHYROPSALIDOIDEA**												

Ischyropsalididae		*Ischyropsalis kollari*	□	□	□	□	□	□	□	□	□	□
Sabaconidae		*Hesperonemastoma modestum*	■	■	□	□	□	■	□	□	□	□
		*Sabacon* sp.	?	?	?	?	?	?	?	?	?	?
		*Taracus* sp.	□	□	□	□	□	□	□	□	□	□

* New subspecies of the Nemastoma bidentatum-group (description in preparation: Tone Novak, personal communication)

NQ, (1,4-naphthoquinone); MNQ, (6-methyl-1,4-naphthoquinone); MOQ, (2-methoxy-1,4-naphthoquinone); MMOQ, (methoxy-methyl-1,4-naphthoquinone); ND, (1,4-naphththalendione); CNQ, (chloro-naphthoquinones); MA, (2-methyl-9,10-anthraquinone); DMAQ, (1,2-dimethyl-9,10-anthraquinone); BQ, (1,4-benzoquinone); ABQ, (alkyl-substituted benzoquinones). ■= detected, □= not detected, ◩= compounds were found in a part of the analyzed species only, ? = no conclusive data.

**Table 4:** Naphtho- and benzoquinone distribution across Laniatores and Cyphophthalmi (metadata*).

Order	(Super)family	*Species*	NQ	MNQ	MOQ	MMOQ	ND	CNQ	MAQ	DMAQ	BQ	ABQ
**CYPHOPHTHALMI**												

	Sironidae	*Cyphophthalmus duricorius*	■	■	□	□	□	■	□	□	□	□
		*Siro exilis*	■	■	□	□	□	■	□	□	□	□
	Pettallidae	*Austropurcellia forsteri*	■	■	□	□	□	■	□	□	□	□
	Stylocellidae	*undetermined species*	■	■	■	□	■**	□	□	□	□	□

**LANIATORES**												

Insidiatores	Travunioidea	*13 species*	□	□	□	□	□	□	□	□	□	□
	Triaenonychoidea	*7 species*	□	□	□	□	□	□	□	□	□	□
Grassatores	Phalangodidae	*2 species*	□	□	□	□	□	□	□	□	□	□
	Stygnommatidae	*1 species*	□	□	□	□	□	□	□	□	□	□
	Stygnopsidae	*2 species*	□	□	□	□	□	□	□	□	□	□
	Manaosbiidae	*2 species*	□	□	□	□	□	□	□	□	□	◩
	Cosmetidae	*more than 5 species*	□	□	□	□	□	□	□	□	□	◩
	Gonyleptidae	*more than 30 species*	□	□	□	□	□	□	□	□	□	◩

* Ekpa et al. (1984), Hara et al. (2005), Raspotnig et al. (2005, 2011, 2012), Gnaspini and Hara (2007), Jones et al. (2009), Shear et al. (2010, 2014), Rocha et al. (2013).

** The naphthalenedione in the undetermined stylocellid was identified as 6-methyl-1,4-naphthalendione (Jones et al., 2009).

NQ, (1,4-naphthoquinone); MNQ, (6-methyl-1,4-naphthoquinone); MOQ, (2-methoxy-1,4-naphthoquinone); MMOQ, (methoxy-methyl-1,4-naphthoquinone); ND, (1,4-naphththalendione derivative); CNQ, (chloro-naphthoquinones); MAQ, (2-methyl-9,10-anthraquinone); DMAQ, (1,2-dimethyl-9,10-anthraquinone); BQ, (1,4-benzoquinone); ABQ, (alkyl-substituted benzoquinones). ■= detected, □= not detected, ◩= compounds were found in a part of the analyzed species only, ? = no conclusive data.

## Abbreviations

BQ: 1,4-benzoquinone
CNQ: 4-chloro-1,2-naphthoquinone
DMAQ: 2,3-dimethyl-9,10-anthraquinone
MAQ: 2-methyl-9,10-anthraquinone
MMOQ: 2-methoxy-6-methyl-1,4-naphthoquinone
MNQ: 6-methyl-1,4-naphthoquinone
MOQ: 2-methoxy-1,4-naphthoquinone
ND: 1,4-naphthalendiol
NQ: 1,4-naphthoquinone
