# Supplementary material for: A Model for Phylogenetic Chemosystematics: Evolutionary History of Quinones in the Scent Gland Secretions of Harvestmen
Source: Front Ecol Evol. Author manuscript; Available in PMC 2018 Mar 9. (PMC5844456; doi:10.3389/fevo.2017.00139)
Supplement: Supplementary Table 1 [file NIHMS76369-supplement-Supplementary_Table_1.docx]

Supplementary Material: Collection sites of the species herein investigated.

| suborder | subfamily | *species* | collection sites (coordinates) |
| --- | --- | --- | --- |
| **Eupnoi**  Phalangiidae | Oligolophinae | *Lacinius dentiger* | **1)** **AUT**: Styria, Deutschlandsberger Klause (46.810073, 15.201355). **2)** **AUT**: Styria, Engelgasse (47.073774, 15.457458). **3) AUT**: Carinthia, Ferlach (46.525848, 14.319031). **4) AUT:** Burgenland, Gloriette Warte (47.854273, 16.504895). **5) AUT:** Styria, Beethovenstraße (47.075713, 15.450613). **6) AUT:** Styria, Einsiedelei (47.076135, 15.388005). **7) AUT:** Styria, Raiffeisenstraße (47.04693, 15.452088). **8) AUT:** Carinthia, Rauth-Moatsche (46.525163, 14.326657). **9) AUT:** Styria, Rosenhain (47.083919, 15.451031). **10) AUT:** Styria, Schubertstraße (47.078257, 15.45374). **11) AUT:** Styria, Universitätsplatz (47.077128, 15.450945). |
|  |  | *Lacinius ephippiatus* | **1) AUT:** Styria, Graz, Rielteiche (47.118239, 15.417812). **2) AUT:** Styria, Rosenhain (47.083919, 15.451031). **3) POL:** Sanok surroundings. |
|  |  | *Lacinius horridus* | **1) AUT:** Burgenland, Gloriette Warte (47.854273, 16.504895). **2) AUT:** Styria, Leibnitz surroundings. **3) AUT:** Styria, Lödersdorf surroundings. |
|  |  | *Mitopus morio* | **1) AUT:** Styria, Lassing, Blosen (47.517027, 14.272837). **2) AUT:** Styria, Deutschlandsberger Klause (46.810073, 15.201355). **3) AUT:** Styria, Murau, Eselsberg (47.223343, 14.203048). **4) AUT:** Carinthia, Ferlach-Osce (46.525848, 14.319031). **5) AUT:** Styria, Gleinalm. **6) AUT:** Carinthia, Gamsgrube (47.088611, 12.735278). **7) AUT:** Styria, Hilmteich (47.084209, 15.46198). **8) AUT:** Styria, Einsiedelei (47.076135, 15.388005). **9) AUT:** Styria, Koralpe. **10) AUT:** Styria, Feldbach, Kornberger Teiche (46.971389, 15.868889). **11) AUT:** Lower Austria, Mönichkirchen-Schwaig. **12) AUT:** Styria, Plabutsch (47.086667, 15.399444). **13) AUT:** Styria, Rosenhain (47.083919, 15.451031). **14) AUT:** Styria, St. Lorenzen (46.664916, 15.183499). **15) AUT:** Styria, St. Oswald, Eibiswald (46.683, 15.100). **16) SLO:** Tivolipark, Ljubljana (46.054395, 14.492299). |
|  |  | *Oligolophus tridens* | **1) AUT:** Styria, Lassing, Blosen (47.517027, 14.272837). **2) AUT:** Styria, Engelgasse (47.073774, 15.457458). **3) AUT:** Styria, Einsiedelei (47.076135, 15.388005). **4) AUT:** Styria, Kroisbach (47.091815, 15.466797). **5) AUT:** Styria, Platte (47.112993, 15.469732). **6) AUT:** Styria, Rosenhain (47.083919, 15.451031). |
|  | Opilioninae | *Egaenus convexus* | **1) AUT:** Styria, Gratkorn. **2) AUT:** Styria, Hilmteich (47.084209, 15.46198). **3) AUT:** Styria, Peggau, Lurgrotte (47.215925, 15.343289). **4) AUT:** Styria, Murufer (47.085012, 15.425438). **5) AUT:** Styria, Platte (47.112993, 15.469732). **6)** **AUT:** Styria, Ragnitzbach/Berliner Ring (47.076949, 15.494955). **7) AUT:** Styria, Rielteiche (47.118239, 15.417812). **8) AUT:** Styria, Rosenhain (47.083919, 15.451031). |
|  |  | *Opilio canestrinii* | **1) AUT:** Styria, Einsiedelei north (47.07688, 15.385374). **2) AUT:** Styria, Engelgasse (47.073774, 15.457458). **3) AUT:** Styria, Beethovenstraße (47.075713, 15.450613). **4) AUT:** Styria, Einsiedelei south (47.076135, 15.388005). **5) AUT:** Styria, Rosenhain (47.083919, 15.451031). |
|  |  | *Opilio dinaricus* | **1)** **AUT:** Burgenland, Gloriette Warte (47.854273, 16.504895). **2) AUT:** Styria, Rosenhain (47.083919, 15.451031). |
|  |  | *Opilio ruzickai* | **1)** **AUT:** Styria, Einsiedelei (47.076135, 15.388005). **2) AUT :** Styria, Rosenhain (47.083919, 15.451031). |
|  |  | *Opilio saxatilis* | **1) AUT:** Styria, Engelgasse (47.073774, 15.457458). **2) AUT:** Styria, Rosenhain (47.083919, 15.451031). |
|  | Phalangiinae | *Phalangium opilio* | **1) AUT:** Styria, Engelgasse (47.073774, 15.457458). **3) AUT:** Carinthia, Ferlach-Osce (46.525848, 14.319031). **4) AUT:** Styria, Hilmteich (47.084209, 15.46198). **5) AUT:** Styria, Einsiedelei (47.076135, 15.388005). **6) AUT:** Styria, Listzgasse (47.044417, 15.453362). **7) AUT:** Styria, Lödersdorf surroundings. **8) AUT:** Carinthia, Malta surroundings. **9) AUT:** Styria, Plabutsch (47.086667, 15.399444). **10) AUT:** Styria, Platte (47.112993, 15.469732). **11) AUT:** Carinthia, Rauth-Moatsche (46.525163, 14.326657). **12) AUT:** Styria, Rosenhain (47.083919, 15.451031). |
|  |  | *Rilaena triangularis* | **1) AUT:** Styria, Lassing, Blosen (47.517027, 14.272837). **2) AUT:** Styria, Deutschlandsberger Klause (46.810073, 15.201355). **3) AUT:** Styria, Feistritzufer (47.080259, 15.986234). **4) AUT:** Burgenland, Gloriette Warte (47.854273, 16.504895). **5) AUT:** Styria, Hieflau (47.609177, 15.469732). **6) AUT:** Styria, Hilmteich (47.084209, 15.46198). **7) AUT:** Styria, Einsiedelei (47.076135, 15.388005). **8) AUT:** Styria, Kroisbach (47.091815, 15.466797). **9) AUT:** Styria, Maria Trost (47.108768, 15.493796). **10) AUT:** Carinthia, Osce (46.525848, 14.319031). **11) AUT:** Styria, Plabutsch (47.086667, 15.399444). **12) AUT:** Styria, Platte (47.112993, 15.469732). **13) AUT:** Styria, Ragnitzbach/Berliner Ring (47.076949, 15.494955). **14)** **AUT:** Carinthia, Rauth (46.523869, 14.327684). **15) AUT:** Carinthia, Moatsche (46.525163, 14.326657). **16) AUT:** Styria, Rielteiche (47.118239, 15.417812). **17) AUT:** Styria, Rosenhain (47.083919, 15.451031). **18) AUT:** Styria, Wagna surroundings. **19) AUT:** Styria, Weizbach (47.113364, 15.459385). |
|  | Platybuninae | *Lophopilio palpinalis* | **1) AUT:** Styria, Feistritzufer (47.080259, 15.986234). **2) AUT:** Styria, Kroisbach (47.091815, 15.466797). **3) AUT:** Styria, St. Oswald, Eibiswald (46.683, 15.100). |
|  |  | *Megabunus armatus* | **1) ITA:** Falzarego (46.51559, 12.02853). **2) ITA:** Grödner Joch (46.55984, 11.81686). **3) AUT:** Tyrol, Karlsbader Hütte (46.76241, 12.79954). **4) SLO:** Kransjka Gora (46.43990, 13.73892). **5) ITA:** Passo di Rolle (46.26603, 11.83746). **6) ITA:** Peitlerkofel (46.66146, 11.81880). **7) SLO:** Prisojnik, Julian Alps (46.42675, 13.75425). **8) ITA:** Lago del Predil, not specified. **9) ITA:** Cima Catinaccio, Rosengarten (46.44016, 11.61334). **10) ITA:** Schlern, Mahlknechthütte (46.49559, 11.64431). **11) ITA:** Schlern, Murmeltierhütte (46.51399, 11.70207). **12) AUT:** Tyrol, Thörl (46.75539, 12.79656). **13)** **AUT:** Carinthia, Trogkofel (46.57316, 13.22012). **14) AUT:** Carinthia, Vellacher Kotschna (46.388889, 14.574167). **15) ITA:** Zoldo Alto (46.39855, 12.06427). |
|  |  | *Megabunus bergomas** | **1) ITA:** Cima di Valmora (45.93114, 9.84190). **2) ITA:** Cimone della Bagozza (46.01371, 10.25145). **3) ITA:** Corna Piana (45.93975, 9.81123). **4) ITA:** Forcella di Valmora (45.93385, 9.83359). **5) ITA:** Olte di Colle (46.54281, 11.80562). **6) AUT:** Tyrol, East Tyrol, not specified. **7) ITA:** Passo di Varicola (45.98516, 10.18002). **8) ITA:** Pass via Rifugio Laeng (45.97673, 10.18933). **9) ITA:** Passo Vivione (46.013711, 10.251444). **10) ITA:** Pizzo Arera (45.928333, 9.804722). **11) ITA:** Pizzo della Presolana (45.96225, 10.06239). **12) ITA:** direction to Pizzo Arera (45.93226, 9.81137). **13) ITA:** Rifugio Albani (45.962256, 10.062394). **14) ITA:** Valle di Arera (45.92837, 9.80232). |
|  |  | *Megabunus leserti* | **1) AUT:** Styria, Admont (47.63395, 14.4516). **2) GER:** Ammergebirge (47.55315, 10.827117). **3) AUT:** Tyrol, Axamer Lizum (47.181167, 11.311694). **4) AUT:** Tyrol, Bayreuther Hütte (47.44513, 11.81491). **5) ITA:** Brenner, Wolfendorn (46.99132, 11.52124). **6) AUT:** Styria, Dachstein (47.516367, 13.677283). **7) AUT:** Styria, Gesäuse, Buchstein (47.604722, 14.592222). **8) AUT:** Styria, Gesäuse, Hochzinödl. **9) AUT:** Styria, Hochschwab, Aflenzer Staritzen (47.631667, 15.270861). **10) GER:** Hoher Gröll (47.604983, 13.067917). **11) GER:** Kampenwand (47.75620, 12.36726). **12) AUT:** Lower Austria, Lunz (47.8097, 15.0779). **13) AUT:** Tyrol, Muttekopfhütte (47.263283, 11.669567). **14) AUT:** Lower Austria, Ötscher (47.866633, 15.219167). **15) AUT:** Styria, Pitzingmoos, Sandling (47.668611, 13.717222). **16) AUT:** Carinthia, Pöllatal, Lieserursprung (47.066389, 13.4525). **17) AUT:** Salzburg, Schafberg (47.7713, 13.422283). **18) AUT:** Lower Austria, Schneeberg (47.786167, 15.807483). **19) AUT:** Styria, Totes Weib (47.734417, 15.482583). |
|  |  | *Megabunus rhinoceros** | **1) ITA:** Breuil-Cervinia (45.944833, 7.615017). **2) ITA:** Campiglia Soana (45.559444, 7.528333). **3) CHE:** Eggerhorn 1 (46.39245, 8.178417). **4) CHE:** Eggerhorn 2 (46.388667, 8.177517). **5) CHE:** Grand-Saint-Bernhard-Tunnel (45.898983, 7.198383). **6) ITA:** Gressoney la Trinité (45.819133, 7.827783). **7) CHE:** Heiligkreuz-Ritterpass (46.3261, 8.172217). **8) CHE:** La Gonillier (46.05125, 7.4919). **9) ITA:** Lys-Glacier, entry of the valley (45.8729, 7.81175). **10) ITA:** Lys-Glacier, terminal moraine (45.885283, 7.820117). **11) CHE:** Mattmark 1 (46.041733, 7.953533). **12) CHE:** Mattmark 2 (46.051683, 7.963433). **13) ITA:** Mont Aric Val di Champ (45.657833, 7.586217). **14) ITA:** Niel near Gaby (45.72555, 7.9161). **15) ITA:** Oropa Lago di Mucrone (45.627317, 7.944). **16) SLO:** Saas-Almagell (46.073408, 13.566042). **17) CHE:** Simplonpass (46.247083, 8.051867). **18) CHE:** Tälliboden (46.003833, 7.976583). **19) ITA:** Valle di Champorcher (45.608817, 7.58875). **20) CHE:** Zinal (46.116567, 7.62775). |
|  |  | *Megabunus vignai** | **1) ITA:** Cima Argentera (44.178889, 7.283056). **2) ITA:** Cima Beccorosso (44.153333, 7.590556). **3) FRA:** Cima de Salauda (44.148183, 7.53405). **4) FRA:** Col de la Bonette (44.347778, 6.78). **5) FRA**: Col de Larche (44.427222, 6.888333). **6) ITA:** Colla della Lombarde (44.219167, 7.141111). **7) CHE:** Eggerhorn 1 (46.39245, 8.178417), **8** CHE, Lämmernsee (46.39425, 7.5921), **9** ITA, Monte Antoroto (44.189167, 7.916111). **10) FRA:** Prarial (44.929444, 6.730556). **11) ITA:** Roc di Fenestrelle (44.160278, 7.3425). **12) ITA:** Val Ellero (44.172617, 7.753267). **13) ITA:** Valdinferno (44.189117, 7.916283). **14) FRA:** Vallon de la Gordolasque (44.097778, 7.395). **15) FRA:** Vallon de Valmasque (44.114722, 7.477778). |
|  |  | *Platybunus bucephalus* | **1) AUT:** Styria, Murau, Eselsberg (47.223343, 14.203048). **2) AUT:** Styria, Gleinalm. **3) AUT:** Styria, Koralpe. **4) AUT:** Lower Austria, Mönichkirchen-Schwaig. **5) AUT:** Tyrol, East Tyrol, not specified. **6) AUT:** Styria, St. Lorenzen (46.664916, 15.183499). |
| Protolophidae |  | *Protolophus niger** | **1) USA:** Shasta County (41.061751, -122.359765). |
|  |  | *Protolophus singularis** | **1) USA:** Tulare County (36.45383, -118.82517). |
| *Dicranopalpus* group |  | *Amilenus aurantiacus* | **1) AUT:** Styria, Deutschlandsberger Klause (46.810073, 15.201355). **2) AUT:** Styria, Hieflau (47.609177, 15.469732). **3) AUT:** Styria, Hilmteich (47.084209, 15.46198). **4) AUT:** Styria, Platte, (47.112993, 15.469732). **5) AUT:** Upper Austria, Reichraming (47.8603309, 14.44196260). **6) AUT:** Styria, Rettenbachklamm. **7) AUT:** Carinthia, Villach. |
|  |  | *Dicranopalpus gasteinensis* | **1) AUT:** Carinthia, Gamsgrube (47.088611, 12.735278). |
| Sclerosomatidae | Gyantinae | *Gyas annulatus* | **1) AUT:** Carinthia, Dixer (46.519252, 14.331850). **2) AUT:** Carinthia, Hermagor (46.593222, 13.340028). **3) ITA:** Monte Pasubio. **4)** **AUT:** Carinthia, Rauth (46.525163, 14.326496). **5) POL:** Tylava, Gmina Dukla (49.442776, 21.710150). |
|  |  | *Gyas titanus* | **1) AUT:** Styria, Deutschlandsberger Klause center (46.810833, 15.200278). **2) AUT:** Carinthia, Hohe Tauern 3 (46.987689, 13.258095). **3)** **AUT:** Styria, Wasserloch Klamm (47.699475, 14.874419). |
|  | Leiobuninae | *Leiobunum blackwalli** | **1) GER:** Mainz, Terrassenstraße (49.997505, 8.263772). |
|  |  | *Leiobunum limbatum* | **1) AUT:** Styria, Engelgasse (47.073774, 15.457458). **2) AUT:** Styria, Grazbachkanal (47.063611, 15.434444). **3) AUT:** Styria, Hauenstein (47.121111, 15.4875). **4) AUT:** Styria, Herz-Jesu Kirche (47.069722, 15.455556). **5) AUT:** Styria, Hieflau (47.609177, 15.469732). **6) AUT:** Styria, Einsiedelei (47.076135, 15.388005). **7) AUT:** Styria, Karolinenweg (47.086667, 15.399444). **8) AUT:** Styria, Rosenhain (47.083919, 15.451031). **9) AUT:** Lower Austria, Wienerwald (48.078889, 16.233333). |
|  |  | *Leiobunum roseum* | **1) AUT:** Carinthia, Eggerloch (46.591667, 13.818333). **2) AUT:** Carinthia, Hermagor (46.593222, 13.340028). **3) ITA:** Northern Italy, not specified. **4) AUT:** Carinthia, Trögener Klamm (46.460833, 14.503889). |
|  |  | *Leiobunum rotundum* | **1) AUT:** Styria, Augartensteg (47.060556, 15.434722). **2) AUT:** Styria, Engelgasse (47.073774, 15.457458). **3) AUT:** Styria, Grazbachkanal (47.063611, 15.434444). **4) AUT:** Styria, Kalvarienbrücke (47.086944, 15.424167). **5) AUT :** Styria, Karolinenweg (47.086667, 15.399444). **6) AUT:** Styria, Laßnitzbrücke (46.794444, 15.530556). **7) AUT:** Carinthia, Malta. **8) AUT:** Styria, Murufer (47.085012, 15.425438). **9) AUT:** Styria, Rosenhain (47.083919, 15.451031). |
|  |  | *Leiobunum rupestre* | **1) AUT:** Styria, Deutschlandsberger Klause (46.810073, 15.201355). **2) AUT:** Styria, Deutschlandsberger Klause center (46.810833, 15.200278). **3) AUT:** Styria, Ebneralm, Johnsbach, (47.525489, 14.651892). **4) AUT:** Styria, Einsiedelei (47.076135, 15.388005). **5) AUT:** Styria, Karolinenweg (47.086667, 15.399444). **6) AUT:** Carinthia, Malta. **7) AUT:** Carinthia, Rauth (46.525163, 14.326496). **8) GER:** Ruhpolding (47.703408, 12.566042). **9) GER:** Unterwössen (47.728333, 12.512778). |
|  |  | *Leiobunum subalpinum* | **1) AUT:** Styria, Deutschlandsberger Klause center (46.810833, 15.200278). **2) AUT:** Carinthia, Hohe Tauern 1 (46.820833, 13.2575). **3) AUT:** Carinthia, Hohe Tauern 2(46.985, 13.231667). **4) AUT:** Styria, Gesäuse, Kodoralm. **5) AUT:** Styria, Koralpe, Oberfresen 1 (46.757222, 15.099167). **6) AUT:** Styria, Koralpe, Oberfresen 2 (46.756944, 15.109167). **7)** **AUT:** Styria, Salzstiegl 1 (47.125556, 14.924722). **8) AUT:** Styria, Salzstiegl 2 (47.0475, 14.924722). **9) AUT:** Styria, Salzstiegl West, not specified. |
|  |  | *Leiobunum sp.^a)^* | **1) GER:** Mainz, Industriehafen (50016667, 8.233333). |
|  |  | *Nelima sempronii* | **1) AUT:** Carinthia, Dixer (46.519252, 14.331850). **2) AUT:** Burgenland, Gloriette Warte (47.854273, 16.504895). **3) AUT:** Styria, Einsiedelei (47.076135, 15.388005). **4) AUT:** Styria, Rosenhain (47.083919, 15.451031). **5) AUT:** Styria, Tautendorf (47.026157, 15.993390). |
|  |  | *Nelima troglodytes** | **1) HRV:** Croatia, not specified. |
|  | Sclerosomatinae | *Astrobunus dinaricus** | **1) MNE:** Tivat, Lepetane (42.460033, 18.685992). |
|  |  | *Astrobunus helleri* | **1) AUT:** Carinthia, Graschelitzen (46.576389, 13.8325). **2) AUT:** Carinthia, Villach surroundings. |
|  |  | *Astrobunus kochi** | **1) FRA:** Mercantour surroundings. |
|  |  | *Astrobunus laevipes* | **1) AUT:** Styria, Lassing, Blosen (47.517027, 14.272837). **2) AUT:** Styria, Gosdorf surroundings. **3) AUT:** Styria, Murpromenade (47.067222, 15.435278). **4) AUT:** Styria, St. Peter surroundings. **5) SLO:** Tivolipark, Ljubljana (46.054395, 14.492299). **6**) **GER:** Mainz, Industriehafen (50.016667, 8.233333). |
| **Dyspnoi**  Dicranolasmatidae |  | *Dicranolasma scabrum* | **1) AUT:** Burgenland, Gloriette Warte (47.854273, 16.504895). |
|  |  | *Dicranolasma soerensii* | **1) FRA:** Llauro surroundings, site 1. **2) FRA:** Llauro surroundings, site 2. |
| Nemastomatidae | Nemastomatinae | *Carinostoma carinatum* | **1) AUT:** Carinthia, Eichholzgraben (46.633333, 13.833333). **2) AUT:** Carinthia, Villach, Graschelitzen (46.576389, 13.8325). **3) SLO:** near Podplat, Rogatec (46.24292, 15.57964). **4) SLO:** Poljane pri Podgradu (45.499167, 14.112222). **5) AUT:** Carinthia, Rauth (46.523869, 14.327684). **6) BIH:** Srpska, Romanija Mt., Pale, Kadino selo (43.924722, 18.595278). **7) SRB:** Kosmaj,(44.468689, 20.572303). **8) SLO:** Spodnje Laknice (45.934167, 15.194167). **9) SLO:** Tivolipark, Ljubljana (46.054395, 14.492299). |
|  |  | *Carinostoma elegans** | **1) SRB:** Kragujevac (44.017978, 20.879183). **2) MNE:** Monastir Morace. **3) SRB:** Vrsac, Vrsacki breg, (45.120628, 21.371783). |
|  |  | *Carinostoma ornatum** | **1) BIH:** Srpska, Romanija Mt., Pale, Kadino selo (43.924722, 18.595278). **2)** **SRB:** Kosmaj,(44.468689, 20.572303). **3) MKD:** Skopje, Matka, near old church St. Nedela (41.94915, 21.288667). |
|  |  | *Centetostoma sp.** | **1)** **ITA:** Turin surroundings. |
|  |  | *Histricostoma argenteolunulatum** | **1) FRA:** Mercantour surroundings. |
|  |  | *Histricostoma dentipalpe* | **1) GER:** Berchtesgarden, Schneitzlreuth. **2)** **SLO.** Ljubljana (46.105306, 14.518222). **3) SLO:** Tivolipark, Ljubljana (46.054395, 14.492299). **4) AUT:** Carinthia, Malta. **5) AUT:** Carinthia, Petzen. **6) ITA:** Turin surroundings. |
|  |  | *Mediostoma humerale** | **1) GRC:** Greece, not specified. |
|  |  | *Mitostoma chrysomelas* | **1) GER:** Berchtesgarden, Schneitzlreuth. **2) GER:** Mainz, Budeheim Steinbruch. **3) AUT:** Styria, Kroisbach (47.091815, 15.466797). **4) AUT:** Styria, Maria Trost (47.108768, 15.493796). **5) AUT:** Styria, Rosenhain (47.083919, 15.451031). **6) POL:** Tylava , Gmina Dukla (49.442776, 21.710150). **7) AUT:** Styria, Wildon. |
|  |  | *Nemastoma bidentatum* | **1) SLO:** Dragonja Valley. **2) ) SLO:** Poljane pri Podgradu (45.499167, 14.112222). **3) GER:** Sachsen, not specified. **4) GER:** Thüringen, not specified. **5) GER:** Schwanewede, Weserinsel Harriersand (53.330009, 8.498347). |
|  |  | *Nemastoma b. bidentatum* | **1) SLO:** Dolenja. **2)** **AUT:** Carinthia, Mairist (46.749264, 14.399411). **3) AUT:** Carinthia, Maria Rain (46.550161, 14.296448). **4) HRV:** Zagreb, Medventen Horvatore. **5) AUT:** Carinthia, Rauth (46.523869, 14.327684). |
|  |  | *Nemastoma bidentatum ssp nov*^b)^* | **1)** **SLO:** Brezovje. **2) SLO:** Podvrh. **3)** **SLO:** Poljane pri Podgradu (45.499167, 14.112222). **4) SLO:** Rakitovec. **5) SLO:** Skocjan. **6) SLO:** Spodnje Laknice (45.934167, 15.194167). |
|  |  | *Nemastoma b.relictum* | **1) AUT:** Styria, Koralpe. **2) AUT:** Styria, St. Lorenzen (46.664916, 15.183499)**.** **3) AUT:** Carinthia, Tschiernok. |
|  |  | *Nemastoma b. sparsum* | **1) SLO:** Crni. **2) AUT:** Styria, Feistritzufer (47.080259, 15.986234). **3) AUT:** Burgenland, Gloriette Warte (47.854273, 16.504895). **4) AUT:** Styria, Kroisbach (47.091815, 15.466797). **5) AUT:** Styria, Hilmteich (47.084209, 15.46198). **6) AUT:** Styria, Maria Trost (47.108768, 15.493796). **7) AUT:** Styria, Murufer (47.085012, 15.425438). **8) GER:** Niedersachen, not specified. **9) GER:** Schwanewede, Weserinsel Harriersand (53.330009, 8.498347). |
|  |  | *Nemastoma bimaculatum** | **1) GER:** Nordrhein Westfalen, Bonn (50.698719, 7.068796). **2) GER:** Nordrhein Wesftalen, Wachtberg (50.645208, 7.079693). |
|  |  | *Nemastoma dentigerum** | **1) GER:** Mainz, Budeheim Steinbruch. **2) GER:** Mainz, Industriehafen (50.016667, 8.233333). **3) ITA:** Turin surroundings. |
|  |  | *Nemastoma lugubre* | **1) POL:** Tylawa, Gmina Dukla (49.442776, 21.710150). |
|  |  | *Nemastoma schuelleri* | **1**) **AUT:** Salzburg, Kaprun, Sigmund Thun Klamm (47.257932, 12.737773). |
|  |  | *Nemastoma triste* | **1) GER:** Berchtesgarden, Schneitzlreuth. **2) AUT:** Styria, Gleinalm. **3) SLO:** Kosenjak. **4) AUT:** Carinthia, Maltatal. **5) AUT:** Salzburg, Kaprun, Sigmund Thun Klamm (47.257932, 12.737773). **6) AUT:** Carinthia, Tschiernok. |
|  |  | *Paranemastoma bicuspidatum* | **1) AUT:** Styria, St. Oswald (46.683, 15.100). **2) AUT:** Carinthia, Treffling. **3) POL:** Tylawa, Gmina Dukla (49.442776, 21.710150). |
|  |  | *Paranemastoma quadripunctatum* | **1) AUT:** Styria, Hilmteich (47.084209, 15.46198). **2) SLO:** Kosenjak. **3) AUT:** Styria, Kroisbach (47.091815, 15.466797). **4)** **AUT:** Styria, Maria Trost (47.108768, 15.493796). **5) AUT:** Carinthia, Osce ((46.525848, 14.319031). **6)** **AUT:** Tyrol, East Tyrol, not specified. **7) BIH:** Srpska, Romanija Mt., Pale, Kadino selo (43.924722, 18.595278). **8)** AUT: Styria, Aibl (46.683046, 15.221222). **9) HRV:** Zagreb sourroundings. |
|  | Ortholasmatinae | *Dendrolasma dentipalpe** | **1) USA:** Humboldt County (40.8699, -124.0725). |
|  |  | *Dendrolasma mirabile** | **1) USA:** Humboldt County (40.8699, -124.0725). |
|  |  | *Ortholasma colossus** | **1)** **USA:** Tulare County (36.45383, -118.82517). |
|  |  | *Ortholasma coronadense** | **1) USA:** San Diego, Marian Bear National Park. |
|  |  | *Ortholasma laevipes** | **1) USA:** Tulare County (36.45383, -118.82517). |
|  |  | *Ortholasma rugosum** | **1) USA:** Sonoma County (38.5951, -123.3369). |
| Trogulidae |  | *Trogulus tingiformis** | **1) AUT:** Carinthia, Maria Rain (46.550161, 14.296448). |
|  |  | *Trogulus spp.^c)^* | **1) AUT:** Carinthia, Maria Rain (46.550161, 14.296448). **2) AUT:** Carinthia, St. Veit, Mairist (46.749264, 14.399411). **3) SLO:** near Podplat, Rogatec (46.24292, 15.57964). |
| Ischyropsalididae |  | *Ischyropsalis kollari* | **1)** AUT, Styria, Peggau, Lurgrotte (47.215925, 15.343289). |
| Sabaconidae |  | *Hesperonemastoma modestum** | **1 USA:** Orgegon, Curry County, NE Brookings (42.1187, -124.1959). **2) USA:** California, Pelomar Mt, San Diego County (33.327125, -116.889512). **3) USA:** Portland, Lewis & Clark. **4** USA, Homestead Loop. |
|  |  | *Sabacon simoni** | **1) ITA:** Alpi maritime (44.066778, 7.8435). |
|  |  | *Taracus sp.** | **1) USA:** Oregon Care Monument. |
|  |  |  |  |

Legend: AUT, Austria; BIH, Bosnia and Herzegovina; CHE, Switzerland; FRA, France; ITA, Italy; GER, Germany; GRC; Greece; HRV, Croatia; MNE, Montenegro; MKD, Macedonia; SLO, Slovenia; SRB, Serbia; USA, United States of America. * non-Austrian species; ^a)^ Wijnhoven, H., Schönhofer, A.L. & Martens, J. 2007. An unidentified harvestman *Leiobunum* sp. alarmingly invading Europe (Arachnida: Opiliones). *Arachnol. Mitt.* **34**: 27-38. ^b)^ Undescribed *Nemastoma bidentatum* (sub)species-complex from Slovenia (T. Novak, personal communication). ^c)^ undetermined.
