# Supplementary material for: A Model for Phylogenetic Chemosystematics: Evolutionary History of Quinones in the Scent Gland Secretions of Harvestmen
Source: Front Ecol Evol. Author manuscript; Available in PMC 2018 Mar 9. (PMC5844456; doi:10.3389/fevo.2017.00139)
Supplement: Supplementary Table 2 [file NIHMS76369-supplement-Supplementary_Table_2.docx]

Supplementary Material: Detailed data to individuals/populations/species herein investigated.

| suborder | family | subfamily | *species* | specimens | ♀ | ♂ | juvenile | not specified | no. of populations |
| --- | --- | --- | --- | --- | --- | --- | --- | --- | --- |
| **Eupnoi** | Phalangiidae | Oligolophinae | *Lacinius dentiger* | 38 | 18 | 7 | 12 | 1 | 11 |
|  |  |  | *Lacinius ephippiatus* | 11 | 3 | 1 | 0 | 7 | 3 |
|  |  |  | *Lacinius horridus* | 6 | 2 | 1 | 3 | 0 | 3 |
|  |  |  | *Mitopus morio* | 60 | 17 | 14 | 15 | 14 | 16 |
|  |  |  | *Oligolophus tridens* | 27 | 19 | 4 | 1 | 3 | 6 |
|  |  | Opilioninae | *Egaenus convexus* | 450 | 18 | 8 | 2 | 422 | 8 |
|  |  |  | *Opilio canestrinii* | 62 | 26 | 26 | 10 | 0 | 5 |
|  |  |  | *Opilio dinaricus* | 2 | 1 | 1 | 0 | 0 | 2 |
|  |  |  | *Opilio ruzickai* | 3 | 2 | 1 | 0 | 0 | 2 |
|  |  |  | *Opilio saxatilis* | 8 | 4 | 3 | 1 | 0 | 2 |
|  |  | Phalangiinae | *Phalangium opilio* | 38 | 10 | 16 | 11 | 1 | 12 |
|  |  |  | *Rilaena triangularis* | 196 | 37 | 46 | 31 | 82 | 19 |
|  |  | Platybuninae | *Lophopilio palpinalis* | 4 | 1 | 2 | 1 | 0 | 3 |
|  |  |  | *Megabunus armatus* | 97 | 20 | 76 | 0 | 1 | 15 |
|  |  |  | *Megabunus bergomas** | 114 | 38 | 76 | 0 | 0 | 14 |
|  |  |  | *Megabunus lesserti* | 202 | 142 | 39 | 0 | 21 | 19 |
|  |  |  | *Megabunus rhinoceros** | 174 | 87 | 87 | 0 | 0 | 20 |
|  |  |  | *Megabunus vignai** | 65 | 23 | 42 | 0 | 0 | 15 |
|  |  |  | *Platybunus bucephalus* | 13 | 4 | 4 | 4 | 1 | 6 |
|  | Protolophidae |  | *Protolophus niger** | 2 | 0 | 0 | 0 | 2 | 1 |
|  |  |  | *Protolophus singularis** | 1 | 0 | 0 | 0 | 1 | 1 |
|  | *Dicranopalpus* group |  | *Amilenus aurantiacus* | 26 | 11 | 14 | 1 | 0 | 7 |
|  |  |  | *Dicranopalpus gasteinensis* | 1 | 0 | 0 | 1 | 0 | 1 |
|  | Sclerosomatidae | Gyantinae | *Gyas annulatus* | 9 | 0 | 3 | 2 | 4 | 5 |
|  |  |  | *Gyas titanus* | 6 | 1 | 0 | 0 | 5 | 3 |
|  |  | Leiobuninae | *Leiobunum blackwalli** | 1 | 0 | 0 | 0 | 1 | 1 |
|  |  |  | *Leiobunum limbatum* | 80 | 28 | 51 | 1 | 0 | 9 |
|  |  |  | *Leiobunum roseum* | 51 | 18 | 26 | 1 | 6 | 4 |
|  |  |  | *Leiobunum rotundum* | 63 | 33 | 29 | 1 | 0 | 9 |
|  |  |  | *Leiobunum rupestre* | 149 | 54 | 88 | 6 | 1 | 9 |
|  |  |  | *Leiobunum subalpinum* | 82 | 15 | 59 | 0 | 8 | 9 |
|  |  |  | *Leiobunum sp.^a)^* | 13 | 6 | 7 | 0 | 0 | 1 |
|  |  |  | *Nelima sempronii* | 38 | 4 | 17 | 17 | 0 | 5 |
|  |  |  | *Nelima troglodytes** | 3 | 0 | 0 | 0 | 3 | 1 |
|  |  | Sclerosomatinae | *Astrobunus dinaricus** | 1 | 0 | 1 | 0 | 0 | 1 |
|  |  |  | *Astrobunus helleri* | 5 | 2 | 0 | 0 | 3 | 2 |
|  |  |  | *Astrobunus kochi** | 1 | 1 | 0 | 0 | 0 | 1 |
|  |  |  | *Astrobunus laevipes* | 32 | 11 | 4 | 0 | 17 | 6 |
| **Dyspnoi** | Dicranolasmatidae |  | *Dicranolasma scabrum* | 3 | 0 | 3 | 0 | 0 | 1 |
|  |  |  | *Dicranolasma soerensii* | 2 | 1 | 1 | 0 | 0 | 2 |
|  | Nemastomatidae | Nemastomatinae | *Carinostoma carinatum* | 70 | 10 | 20 | 0 | 40 | 9 |
|  |  |  | *Carinostoma elegans** | 50 | 8 | 18 | 0 | 24 | 3 |
|  |  |  | *Carinostoma ornatum** | 32 | 4 | 5 | 0 | 23 | 3 |
|  |  |  | *Centetostoma sp.** | 1 | 0 | 0 | 0 | 1 | 1 |
|  |  |  | *Histricostoma argenteolunulatum** | 2 | 1 | 1 | 0 | 0 | 1 |
|  |  |  | *Histricostoma dentipalpe* | 12 | 0 | 5 | 3 | 4 | 6 |
|  |  |  | *Mediostoma humerale** | 3 | 0 | 0 | 0 | 3 | 1 |
|  |  |  | *Mitostoma chrysomelas* | 24 | 1 | 3 | 0 | 20 | 7 |
|  |  |  | *Nemastoma bidentatum* | 44 | 11 | 1 | 0 | 32 | 5 |
|  |  |  | *Nemastoma b. bidentatum* | 41 | 5 | 2 | 0 | 34 | 5 |
|  |  |  | *Nemastoma bidentatum ssp nov*^b)^* | 76 | 0 | 0 | 0 | 76 | 6 |
|  |  |  | *Nemastoma b.relictum* | 38 | 9 | 15 | 0 | 14 | 3 |
|  |  |  | *Nemastoma b. sparsum* | 120 | 10 | 17 | 6 | 87 | 9 |
|  |  |  | *Nemastoma bimaculatum** | 2 | 0 | 0 | 0 | 2 | 2 |
|  |  |  | *Nemastoma dentigerum** | 31 | 0 | 0 | 0 | 31 | 3 |
|  |  |  | *Nemastoma lugubre* | 4 | 3 | 0 | 0 | 1 | 1 |
|  |  |  | *Nemastoma schuelleri* | 2 | 0 | 0 | 0 | 2 | 1 |
|  |  |  | *Nemastoma triste* | 45 | 0 | 1 | 0 | 44 | 6 |
|  |  |  | *Paranemastoma bicuspidatum* | 9 | 0 | 0 | 0 | 9 | 3 |
|  |  |  | *Paranemastoma quadripunctatum* | 23 | 4 | 8 | 3 | 8 | 9 |
|  |  | Ortholasmatinae | *Dendrolasma dentipalpe** | 3 | 0 | 0 | 0 | 3 | 1 |
|  |  |  | *Dendrolasma mirabile** | 1 | 0 | 0 | 0 | 1 | 1 |
|  |  |  | *Ortholasma colossus** | 4 | 2 | 1 | 0 | 1 | 1 |
|  |  |  | *Ortholasma coronadense** | 6 | 0 | 0 | 0 | 6 | 1 |
|  |  |  | *Ortholasma laevipes** | 4 | 1 | 2 | 0 | 1 | 1 |
|  |  |  | *Ortholasma rugosum** | 1 | 0 | 0 | 0 | 1 | 1 |
|  | Trogulidae |  | *Trogulus tingiformis** | 1 | 0 | 0 | 0 | 1 | 1 |
|  |  |  | *Trogulus sp.^c)^* | 6 | 2 | 0 | 3 | 1 | 3 |
|  | Ischyropsalididae |  | *Ischyropsalis kollari* | 5 | 2 | 1 | 0 | 2 | 1 |
|  | Sabaconidae |  | *Hesperonemastoma modestum** | 27 | 0 | 1 | 0 | 26 | 4 |
|  |  |  | *Sabacon simoni** | 3 | 0 | 0 | 0 | 3 | 1 |
|  |  |  | *Taracus sp.** | 1 | 0 | 0 | 1 | 0 | 1 |
| **total** |  |  |  | **2830 individuals** | **730** ♀ | **858** ♂ | **137 juv** | **1105** | **361** |
|  |  |  |  |  |  |  |  |  |  |

Legend: * non-Austrian species; ^a)^Wijnhoven, H., Schönhofer, A.L. & Martens, J. 2007. An unidentified harvestman *Leiobunum* sp. alarmingly invading Europe (Arachnida: Opiliones). *Arachnol. Mitt.* **34**: 27-38. ^b)^ Undescribed *Nemastoma bidentatum* (sub)species-complex from Slovenia (T. Novak, personal communication). ^c)^ undetermined.
